# A Comprehensive Study on Technologies of Tyre Monitoring Systems and Possible Energy Solutions

**DOI:** 10.3390/s140610306

**Published:** 2014-06-11

**Authors:** Ali E. Kubba, Kyle Jiang

**Affiliations:** 1 Fusion Innovations Ltd, Research and Innovation Services, Birmingham Research Park, Vincent Drive, Edgbaston, Birmingham, B15 2SQ, UK; 2 School of Mechanical Engineering, University of Birmingham, Edgbaston, Birmingham, B15 2TT, UK; E-Mail: k.jiang@bham.ac.uk

**Keywords:** TPMS, pressure sensor, energy harvesting means, tyre structure

## Abstract

This article presents an overview on the state of the art of Tyre Pressure Monitoring System related technologies. This includes examining the latest pressure sensing methods and comparing different types of pressure transducers, particularly their power consumption and measuring range. Having the aim of this research to investigate possible means to obtain a tyre condition monitoring system (TCMS) powered by energy harvesting, various approaches of energy harvesting techniques were evaluated to determine which approach is the most applicable for generating energy within the pneumatic tyre domain and under rolling tyre dynamic conditions. This article starts with an historical review of pneumatic tyre development and demonstrates the reasons and explains the need for using a tyre condition monitoring system. Following this, different tyre pressure measurement approaches are compared in order to determine what type of pressure sensor is best to consider in the research proposal plan. Then possible energy harvesting means inside land vehicle pneumatic tyres are reviewed. Following this, state of the art battery-less tyre pressure monitoring systems developed by individual researchers or by world leading tyre manufacturers are presented. Finally conclusions are drawn based on the reviewed documents cited in this article and a research proposal plan is presented.

## 1. Introduction

This article presents a research proposal plan on developing a functional tyre condition monitoring system (TCMS). The proposed device is planned for land vehicles, but can also be used for aircraft with limited modifications. A TCMS, also called a Tyre Pressure Monitoring System (TPMS), when it is primarily installed to monitor inflation pressure, is an electronic device by which tyre conditions, such as tyre inflation pressure, cavity air temperature, tyre belt stress and strain, normal load, traction force, acceleration, tyre wear, *etc.*, are measured and transmitted to notify the vehicle driver about the values of the measured quantities, the status of the tyre, and to warn the driver if there is any unsafe change in any of the measured values, e.g., low inflation pressure. TCMS is usually installed inside the pneumatic tyre cavity, but it can also be designed to be tightened onto the tyre valve stem cap externally.

Deflated tyres can cause several problems which may lead to insecurity; extra fuel consumption; excessive tyre wear; reduction in tyre lifetime; higher rolling resistance; noise emissions and escalation in CO_2_ emissions. It is estimated that from the introduction of the TPMS to the EU-15 alone; reduction in CO_2_ emissions can be 9.6 million tons per year by 2020. On average; it is estimated that maintaining the recommended tyre inflation pressure can reduce CO_2_ emissions and fuel consumption by about 2.5%. In a passenger car tyre; the recommended inflation pressure lies within 1.9–2.2 bar pressure range. A decrease of 0.5 bar in the recommended inflation tyre pressure may increase that tyre rolling resistance by 10%; leading to an increase in fuel consumption of 2.5% [1]. According to Transense technologies plc; a company based in Bicester; UK; has developed a SAW based tyre pressure monitoring system which is supposed to be available on the market for motorsport applications [2]; a car that has tyres that are 20% underinflated has an increased rolling resistance of 12%; reducing tyre lifetime by up to 50% and leading to increased fuel consumption by 6% [3]. Existing TPMSs require a power supply; which is nearly always a battery; however; an external power supply is possible; e.g., electromagnetic coupling and/or RF power telemetry. Problems with batteries in TPMSs can be summarised into three main categories; safety issues due to battery finite life; environmental impact consequences primarily due to the disposing of toxic chemicals contained in depleted TPMS batteries (Spectrum Batteries Inc.: Flushear, TX, USA) [4]; and initial and then maintenance costs; the former is due to the need for specially made batteries for the TPMS environment; while the latter results from the need for the depleted batteries replacement. A TPMS battery is subjected to the tyre cavity's harsh environment which can significantly deteriorate its efficiency by the extreme climatic conditions inside the tyre; e.g., a wide operation temperature range and battery contacts' problems resulting from the extreme vibration conditions occurring within the tyre-rim assembly [5]. External TPMS powering; whether via electromagnetic coupling or through an RF powered system; relatively consume a high amount of power (Delta Electronics Inc.; Taipei; Taiwan) [6]; which therefore results in the consuming of more fuel. Furthermore; the reader part of these types of tyre monitoring systems has to be installed within a few centimetres of the sensor embedded inside the tyre. The TCMS developed in this research avoids the energy problem associated with conventional TPMSs by using an energy harvesting technique which converts a fraction of the strain energy dissipated throughout the pneumatic tyre cyclic deformation into electrical charge and stores it in an electrical capacitor in order to use it via the system circuitry. In this way; using batteries for powering the TCMS is eliminated and therefore obviates the disposal of a huge amount of depleted batteries; which is a contribution towards eco-driving.

In order to obtain a long life vehicle tyre and to promote road traffic safety, research has been conducted by several researchers and by some of the biggest tyre manufacturers, e.g., Michelin, Continental, Pirelli and Dunlop [7] to produce a means to sustain road traffic safety by improving vehicle control system and tyre-road interaction, e.g., tyre contact patch area and pressure distribution within it , that is an intelligent tyre.

Maintaining tyre pressure levels is particularly vital for avoiding traffic accidents and prevents wasting fuel caused by faulty tyre air pressure levels; which therefore helps the reduction of carbon emissions. In the US, the government has put in to place legislation in which all new vehicles since September 2007, except those with dual wheels on an axle, have to have a TPMS installed in all its tyres. This is enforced by the National Highways and Traffic Safety Administration (NHTSA)-(FMVSS No. 138) [8–15]. Likewise, in the EU from 1st November 2012, all new vehicles must have a TPMS installed (UNECE Vehicle Regulations (Regulation No. 64))[15–17]. The next paragraph explains the problems associated with powering a tyre monitoring system.

The problems associated with using batteries as the main power supply unit in TPMSs include limited life time, low energy density at extreme temperatures [18,19], energy capacity deterioration associated with high centrifugal loading, shocks and vibration exposure, and the need for frequent replacements [20,21]. The technique used in the proposed TCMS is a clean and perpetual solution to powering sensors by avoiding the use and the need for the toxic and corrosive thionyl chloride chemistry used in TPMS lithium batteries, and subsequently their resulted waste, in which special handling and disposal is required. However, the designed system is active only when the containing tyre is in motion. This property helps by saving energy from being wasted while the vehicle is stationary and accelerates waking up time after starting off.

**Figure 1.**
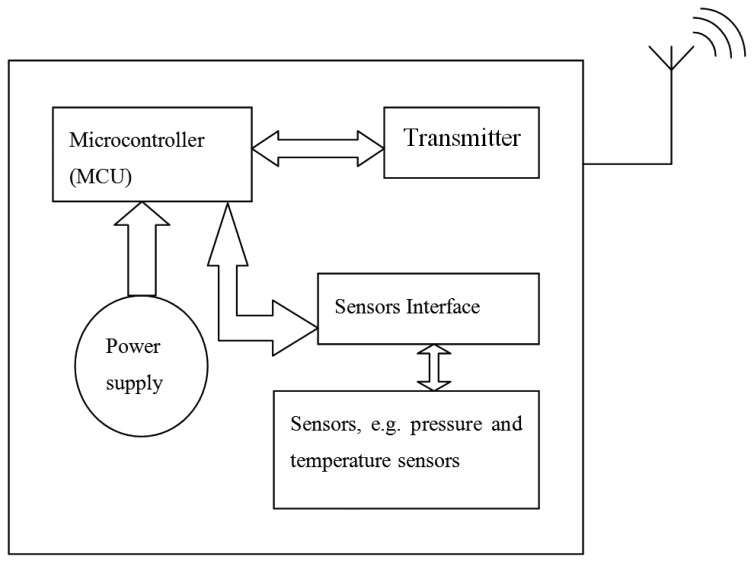
General TCMS schematic diagram.

In general, a TCMS consists of three main units; the power supply unit, sensing unit, and readout circuitry unit as shown in Figure 1. The power supply unit comprises an energy source element which is primarily a battery, and a power monitoring and conditioning circuitry. The sensing unit includes different types of sensors with respect to the properties intended to be measured, e.g., pressure and temperature sensors. The third unit is a programmable element run by a microcontroller. Its function is summarised into handling the system sensors' input and output signals using an interface circuit, switching on and off the system components, and transmitting the measured quantities [22–24].

The research is planned to address the power requirements and sensing mechanism issues related to the above mentioned three main TCMS units in such a way that an energy harvester is installed on the inner surface tyre linear. In addition, the overall power consumption of the system must be within the energy harvester power generation capacity in order to achieve an acceptable duty cycle. A duty cycle is the fraction of the time over which a device is active divided by the length of a certain period of time.

**Figure 2.**
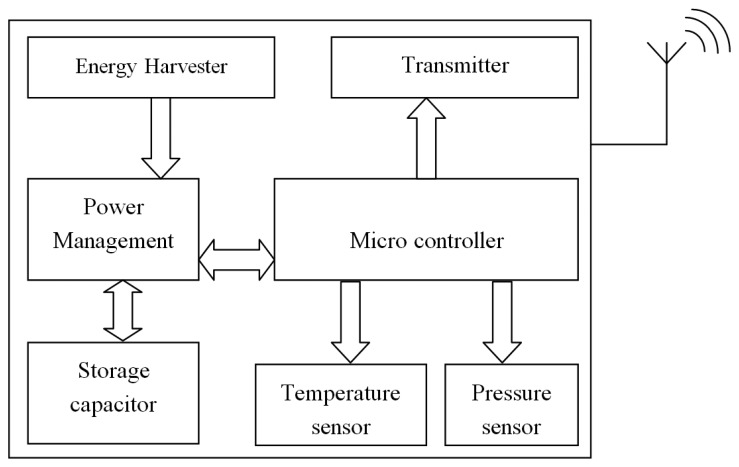
TCMS schematic diagram.

Figure 2 shows the developed TCMS in this research. The main difference in this system, compared to the general TCMS components, is the power supply unit. This device is self-powered and therefore does not require using a battery or any other means of contact free powering methods, e.g., induction coupling. The research is intended to develop a TCMS that can be attached to the inside of a passenger vehicle pneumatic tyre. Having the system applied on that area allows the sensing of additional parameters rather than inflation pressure and tyre cavity air temperature, e.g., traction force, normal load, tyre strain, acceleration, *etc.* and promotes the intelligent tyre system.

The key feature of the TCMS developed in the research is its energy scavenging capability and wide speed range coverage. Instead of embedding batteries to power sensors and the transmitter, it converts tyre strain energy into electricity using a piezoelectric device. The developed TCMS has the advantages of minimal maintenance requirements, obviating the disposal of large quantities of batteries, and the compatibility of functioning over an automotive temperature range of a higher level than lithium batteries.

The development of the energy harvesting based TCMS went through logical steps as follows. First, a micro tyre pressure sensor and several types of electronic circuits for both power management, and measuring and transmitting the embedded sensors data purposes were designed and tested. Then two energy harvesting units were studied. The harvesting unit of the higher energy density was then selected for pneumatic tyre application. This energy harvesting unit which can operate efficiently in the tyre cavity atmosphere conditions and generates sufficient energy for the TCMS circuitry was promoted by testing its performance via applying it inside a pneumatic tyre and observing its performance under different loading and speed conditions. Experiments were then conducted to examine the performance of the selected Piezo Fibre Composite (PFC) energy harvester in the School of Mechanical Engineering laboratory by using a tyre test rig, in which controlled loading and speed conditions were applied. Then the chosen energy harvester, coupled with the integrated TCMS circuitry including the pressure and temperature sensors, were tested on both the tyre test rig and on the road including motorways to validate the designed TCMS performance and to make it ready for application in passenger vehicle tyres.

The article structure starts with a historical review of pneumatic tyres and means to measure tyre inflation pressure, followed by a comparison between major pressure sensing techniques. Then possible extractable energies and major energy extraction techniques within rolling tyres are illustrated in detail. A state of the art of useful energy harvesting techniques and ones developed by researchers for powering TPMS is presented. Following that power conditioning and TCMS design principles are explained. Finally conclusions and discussion in order to establish a research plan for designing a TCMS.

## 2. TCMS Design Principles

The aim of the research proposal plan is to design and develop a competitive battery-free tyre condition monitoring system (TCMS) and to test its feasibility in the laboratory and on the road. The power source is aimed to be a robust and durable energy harvesting unit that can resist tyre cavity conditions and is able to perform up to standard over a wide range of vehicle speeds and loading conditions for the lifetime of the tyre. An additional aim is to design a novel low power micro pressure sensor as a part of the TCMS to reduce the overall system power requirement, particularly when tyre air pressure readings are being taken. It is also intended that the developed TCMS will have a miniature structure suited for installation on the inner tyre surface.

## 3. Historical Review

This section summarises how the pneumatic tyre was developed over history and how different methods have been used to measure tyre inflation pressure.

### 3.1. Pneumatic Tyre

Tyre specification is always a matter of selecting the most appropriate design based on a number of inter-related and often conflicting properties. The first documented invention of the pneumatic tyre was in 1845 by Robert William Thomson, who called the invention the Aerial Wheel. This invention introduced three new advantages of the Aerial Wheel: reduced rolling resistance, better manoeuvrability, and decreased noise while in motion. At that time, Thomson first suggested that the new tyre would be inflated with air along with “various solid substances of an elastic quality”. A tyre with multiple tubes was first developed. Wired type tyre construction with a straight side rim appeared in 1922 made by Dunlop. Back in 1892, a tubeless tyre was predicted to be the future by W. H. Paull. It took approximately six decades until tubeless tyres became commercially available [25]. The next section summarises the development of air pressure sensors following the discovery of pressure phenomena by Galileo in 1594.

### 3.2. Air Pressure Measurement

Having pneumatic tyres filled with air, makes the air act as an ideal compressible elastic support for the tyre structure and the amount of air needed inside the tyre is directly determined by the vertical load capacity for which the tyre is designed. Friction coefficients, both static and kinetic, are mildly affected by inflation pressure on dry roads, while on wet surfaces inflation pressure is a crucial factor to improve both friction coefficients [26]. Having the correct tyre pressure benefits optimal performances in several land vehicle controls, e.g., braking distance and less tyre wear. It also saves fuel and reduces the possibility of having unexpected tyre break down and overheating, which can be a direct cause of fatal accidents [27,28]. Noise generated from rolling tyres increases when tyres are under low inflation pressure. These facts make measuring tyre air pressure vital and therefore different types of tyre pressure measurement have been developed. A detailed report by W. Reithmaier *et al.* [29] demonstrates and analyses tyre-related accident research data and summarises the main effects of under inflated tyres on their durability and overall vehicle performance. In addition, this report presents different techniques and methods to monitor, control and maintain tyre inflation pressure.

Mechanical pressure gauges are the most common tyre pressure measurement tool and still the most widely used instrument for measuring pressure of different fluids. Originally they were invented for measuring atmospheric pressure, as the aneroid barometer by Lucien Vidie for low pressure in 1844, and 5 years later developing into the Bourdon tube pressure gauge for high pressures [30–32]. These pressure sensors were the first ones that did not use liquids for the pressure measurement mechanism; instead they use mechanical linkages, springs, a diaphragm, and bellows to detect any applied pressure and result in corresponding movement.

In 1856, Lord Kelvin discovered the piezoresistive effect in metals which was an essential factor in developing strain gauges [33]. Electrical pressure transducers were developed in the early 1930s by applying potentiometers or variable capacitors on a Bourdon tube. In the late 1930s, pressure transducers were developed using strain gauges by applying them on pressure sensitive diaphragms after the invention of independent bonded strain gauges by E. E. Simmons of the California Institute of Technology and AC. Ruge of Massachusetts Institute of Technology [34]. In the mid-1950s, the piezoresistive effect in semiconductors, which is comparatively much higher than its parallel in metals, was found by C. S. Smith, and since then, various kinds of micromachined sensors, including micro pressure sensors, have been widely produced [33,35]. Having strain gauges bonded to a pressure sensitive diaphragm causes inescapable hysteresis and instability. Thin-film transducers with good stability and low hysteresis were introduced in the 1960s by Statham. This technology is particularly useful for high pressure sensors' fabrication. As thin-film technology is more suitable for high pressure application, William R. Poyle applied for a patent for a capacitive transducer on glass or quartz basis in 1973, whereas Bob Bell of Kavlico did the same on ceramic basis in 1979 [36].

The automotive industry has been using silicon micromachining since the mid-1970s where primarily pressure sensors were applied to monitor and control air-fuel ratio to improve fuel economy. Since the 1990s micro pressure sensors were applied to detect fuel vapour leak to reduce raw fuel emissions [37].

**Figure 3.**
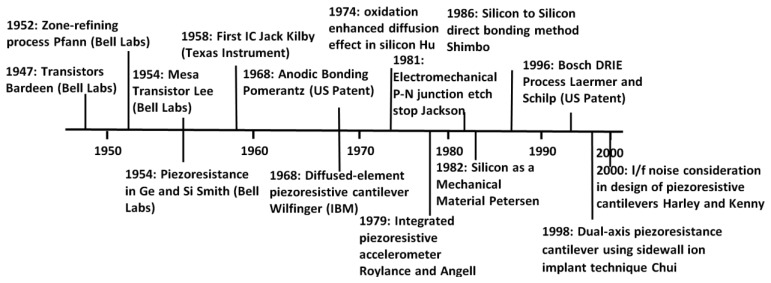
Technological advances in IC fabrication (above the horizontal line) and micromachining (below the horizontal line) [38].

Having MEMS pressure sensors mass produced with a relatively low unit cost, small weight, micro size and higher precession than conventional pressure sensors, opened a huge market in the automotive industry and made them practical for various pressure related automotive applications. In addition, technological advances in the fabrication of integrated circuits (IC) including doping, etching, and thin film deposition methods, have allowed significant improvements in piezoresistive device sensitivity, resolution, bandwidth, and miniaturization [38]. The relation between ICs and MEMS pressure sensors' micro fabrication evolution over virtually the second half of the 20th century is demonstrated in Figure 3. The next section compares different types of pressure sensing mechanisms within MEMS scale, among which some might be suited for tyre pressure monitoring systems.

## 4. MEMS Tyre Pressure Sensor

**Figure 4.**
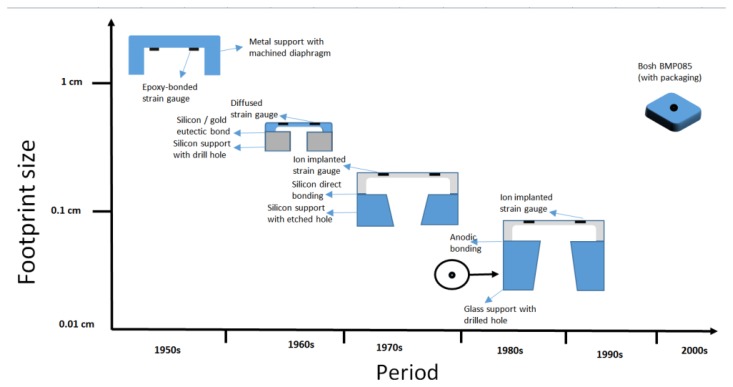
The evolution of diaphragm-based MEMS pressure sensor [38].

Macroscopic pressure sensors used for detecting pressure greater than atmospheric pressure share a common characteristic with deformable diaphragms, and they are also the case with their micro scale counterparts [35]. Diaphragms are the simplest mechanical structure for pressure sensing and have been employed in micro machined pressure sensors because of their compatibility with a range of bulk and surface silicon micromachining processes [39]. The deflection of the pressure sensitive diaphragm can be detected by either measuring the diaphragm deflection or by measuring the stresses produced in the diaphragm [39,40]. Diaphragms can take different geometries depending on their purpose and whether there is a stress or deformation limitation in that application [41,42]. Figure 4 shows the evolution of diaphragm-based MEMS pressure sensors.

**Figure 5.**
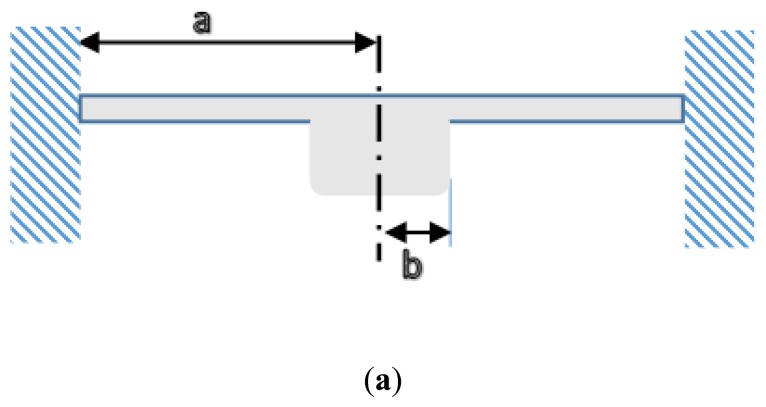

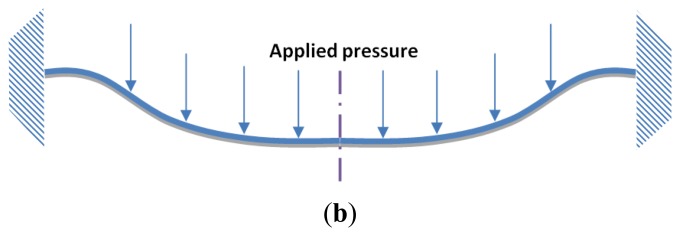
(**a**) Bossed diaphragm geometry and (**b**) its associated displacement under uniform pressure [39].

Bossed diaphragms, as shown in Figure 5, are attractive in the case of a traditional bonded strain gauge pressure sensor as they offer mechanical stress amplification.

When pressure is applied on a bossed diaphragm, the developed stresses on the inner and the outer perimeters are the greatest and it is higher than the maximum which occurs in a flat diaphragm [39]. This type of diaphragm offers more sensitivity and linearity in pressure-capacitance response. However, they are more suited for low pressure detection sensors [35,39,43]. In addition, and particularly in TPMS application, having a bossed diaphragm causes considerable error in pressure measurement due to the high centrifugal force up to 3000 g [23], or shocks from the road up to 1000 g [27].

In general, diaphragm-based micro machined pressure sensors can be classified, according to the pressure sensing principle, into piezoresistive, capacitive and resonant pressure sensors. These three most common types of MEMS pressure sensors in automotive application, particularly with tyre inflation pressure, are presented and compared in the following sections. Other types of pressure sensors like piezoelectric and optical pressure sensors are outside of the scope of this research due to the high cost and complexity of the measuring chain associated with handling these types of sensors [44], and also because of their high power consumption, which make them not well suited for TCMS application.

### 4.1. Justification of Focusing A Study on Capacitive Type Pressure Sensors

A comparison between pressure sensors; piezoresistive, capacitive and resonant pressure sensors, the most used microsensors, is illustrated in Table 1.

From Table 1 and the presented literature, it can be seen that capacitive pressure sensors are the least power hungry, which is essential for building a self-powered TCMS. In addition, they have a reasonable temperature sensitivity which is acceptable for the TCMS application.

### 4.2. Effects of Diaphragm Geometry on Pressure Sensors' Performance

Diaphragm based pressure sensors can have various diaphragm shapes, e.g., circular, square or rectangular. Diaphragm geometries react differently when uniform pressure is applied on one side of the diaphragm. Wang and Ko [46] presented a comparison between the three diaphragms shapes' responses under uniformly applied pressure. The diaphragms were compared in two ways; equal areas or equal width. Stress and deflection were simulated using FEA and presented against different applied pressure for all the above mentioned diaphragm shapes. These results show that a circular diaphragm has more advantages over the other diaphragm geometries when diaphragm areas are equal. The lowest maximum stress and the highest sensitivity occur when a circular diaphragm is employed. However, an elliptical diaphragm, with the same area as a circular one, may offer less principal stresses [47,48], in the cost of a slight reduction in sensitivity, in which case extra pressure range and thermal stresses may be covered. Therefore, a comparison between a circular and elliptical diaphragm shape is necessary in terms of obtaining a pressure sensor with acceptable pressure sensitivity, less principal stresses, and low temperature sensitivity.

## 5. Possible Energy Sources for a Tyre Monitoring System

One of the greatest challenges in having an intelligent tyre system is providing the required energy to power the sensors and circuitry included in it. To power such a system, using a battery could be the easiest option and it is the most common way of powering TPMSs. However, the fact that it has a finite life creates maintenance and disposal problems and environmental impact issues, particularly because of the establishment of TPMS legislation in the US and forthcoming in the EU. Furthermore, tyre cavity boundary conditions, such as centrifugal and impact forces, vibration, and extreme temperatures, are negative factors on battery life and performance. For these reasons, extensive research has been carried out in order to find alternative power sources that are feasible for this system [7,21]. Section 0 explains in more detail the state of the art of energy harvesting in tyres.

Primarily, for a battery-less TPMS, there are two types of powering techniques; passive or remotely powered systems, which could be RF powered, an induction loop system or SAW based sensor node, and active or self-powered systems, which scavenge energy from tyre motion and/or deformation. The latter can be achieved by having a micro power generator that can convert either kinetic -vibration- or strain energy -deformation- into electrical energy using different means of transducers' elements, so called harvesters, e.g., piezoelectric, electromagnetic and electrostatic among which piezoelectric harvesters are the highest power density for micro generators as observed in the literature [49–52]. These energy harvesting techniques will be covered briefly and compared in section 0 with focus on the piezoelectric method, as the latter lies within the research thrust area.

The mechanism, upon which an energy harvester relies, varies in nature depending on the ambient energy source. Chris Knight *et al.* [53] presents the state-of-the art technology in both energy harvesting and storage for sensor nodes.

As energy harvesting in vehicle tyres is the application in this research, it will focus on evaluating the main harvestable energy within the vehicle tyres' domain during motion conditions. Other energies, e.g., thermal gradient and air flow energies [54–56], are out of the scope of this research due to the large occupancy requirement associated with these types of energy harvesters and their low power density, which make them not well suited, and less reliable than strain energy harvesting, in a TCMS application.

Tyre cyclic deformation, as a result of rolling on a hard surface, offers three main types of a mechanical energy harvesting mechanism; bending, stretching, and vibration. Bending and stretching occur in the tyre contact patch every tyre revolution. Inner liner tyre strain depends on several factors, e.g., load, position and tyre material properties. Inner tyre liner deformation occurs in cyclic bases, preliminary in the contact patch area, as a result of rolling on hard surfaces. This cyclic deformation offers an attractive source for energy harvesting using piezoelectric materials that transform this strain energy into useful electric energy. Choosing an appropriate piezoelectric material can be tricky, mainly because of the amount of tyre strain and temperature dependent conditions [57,58].

Attempts to harvest energy from direct strain energy in a tyre inner surface were carried out by Apollo [58], [59] and Ende *et al* [57]. In 2005, Apollo achieved a maximum power of 0.9 mW at 80 km/h using a 80 mm × 80 mm layer of PVDF film piezo element under a 400 kg load. The main issues are the durability of the piezoelectric harvester, the maximum allowable strain that the harvester can handle and at which temperature range the piezoelectric can function. It was concluded by the Apollo project team that PVDF is not suitable for direct strain energy harvesting in tyres due to its lack of resistance to tyre temperature and strain effects, and that Micro Fibre Composite (MFC) is a better candidate for the job. In December 2011, Ende *et al* [57] were able to develop a new type of PVDF that can remain relatively stable under high temperature conditions compared to an MFC element (Smart Materials), which showed a significant amount of hysteresis between loading and unloading. The issue with the developed PVDF is its low power generation level, which was estimated to generate a maximum of 30 μW/cm^2^ at 50 km/h by using a (40 × 16.5 mm^2^ of active area) layer of PVDF element (Polyvinylidene fluoride). Due to the viscoelstic nature of the PVDF -piezoelectricity charge coefficient (*d*_33_) value depends on strain rate- and its composite matrices, the power generated at higher speeds is not easy to predict. The results obtained by Ende were based on tyre contact patch length as the main parameter within the range (40–140) mm, loading or inflation pressure have no influence in these analyses.

Cyclic bending within a rolling tyre is another attractive energy harvesting mechanism. In November 2011, Makki [59] presented three energy harvesting techniques within a tyre structure based on tyre deformation in various parts in the tyre, but instead of directly bonding the piezoelectric element to the inner tyre surface, a very thin piezoelectric element was bonded to a reinforcement plate, layer, or ribbon. The first design consisted of a PZT bender bonded to the inner liner of the tyre opposite to the treads; that is a flexible PZT unimorph made of a 25 mm in diameter PZT on a 44 mm diameter plate. The second design was a PVDF bender bonded to the tyre. The latter was composed of a 40 × 40 mm^2^ × 100 μm thick PVDF element bonded to a 0.3 mm thick plastic reinforcement sheet. Both of the first and second designs were bonded on the inner surface of the tyre belt using a very flexible high temperature adhesive to allow the harvester to deform with the tyre while having the least effect on the tyre deformation pattern. The third design contained a PVDF ribbon attached to the tyre bead from side to side; that is a 270 × 20 mm^2^ ribbon with 4 PVDF elements attached to it structurally, and connected in parallel with individual rectification circuits electrically. This design is unlike the first and second designs as it is not directly adhered to the tyre, instead it is based on the deformation occurring in the plastic ribbon due to the changing in tyre section height. Experimental tests showed that the first design had the highest energy harvesting rate among the three designs described. At 80 rpm, the first design generated an average power of 4.6 mW with a peak voltage of 45.5 V. Similarly and under the same motion conditions, the second design generated 0.85 mW with a peak voltage of 62.3 V, and the third design generated 0.23 mW and 18.7 V. Different resistive loads were used for each design to approach the maximum power output condition. It's worth mentioning that in the latter study by Makki[59], the tyre deformation criteria was the contact patch length, which was tested within the range (11.43–16.51) cm, based on an average contact patch length of 14 cm for an average passenger vehicle. The experimental results however showed that the output power has insignificant dependency on the contact patch length, as long as it at least covers the piezoelectric harvester entirely.

As the electric charge generated from the piezoelectric harvester is directly proportional with tyre strain, the value of the generated electric charge can be translated to different quantities. Use of Piezoelectric transducers can be applied to monitor different aspects of tyre conditions, e.g., strain, loading, speed, contact patch dimensions, and friction force. Jingang Yi [60] applied a PVDF transducer on an inner tyre liner to estimate tyre strain by measuring the amount of electric charge generated by the PVDF. In this study, a detailed derivation of the electric charge generated from tyre deformation, for both bending and stretching cases, was presented. However, the derived formulae need special equipment to determine individual tyre properties, e.g., friction coefficient, contact patch length, tyre longitudinal stiffness, and the shear modulus of the tyre carcass [60,61]. Numerical attempts were carried out to estimate tyre deformation under different loading and rolling speed conditions by several researchers [62–66]. However, numerical results are not very accurate and experimental investigation is necessary to determine tyre stress and strain and therefore to estimate the performance of a direct strain energy harvester applied to the inner tyre surface.

Tyre vibration is an attractive energy source in which energy harvesting might be applied. Several studies have been completed to measure tyre vibration under different loading and road surface conditions using different techniques. For instance, the Pirelli Tire System project in co-operation with the Mechanical Engineering Department of the Politecnico di Milano have published a paper regarding measurements of pneumatic tyre acceleration under rolling conditions using a three-axial MEMS accelerometer [67]. From this paper, it can be seen that harvestable vibration energy is around the 100 Hz range. Kindt *et al* [68] carried out experiments on tyre vibration, and collected experimental data using a Laser Doppler vibrometer and the high power vibration energy density was also around 100 Hz. A similar frequency spectrum pattern was obtained by Roundy [20,49] and Löhndorf *et al* [21]. Vibration based piezoelectric, electrostatic and electromagnetic micro generators for tyre pressure monitoring have been developed by several researchers and companies [69–79], but in most cases, micro generator performance highly depends on the applied frequency in such a way that it has a quite narrow band width of the efficient power generation level around its resonance frequency which makes it not suitable for the variable excitation frequency environment, such as in land vehicle tyres. However, vibration energy harvesters can be a good option when applied on constant speed machinery by toning their resonance frequencies with the machines' operation speeds. Khameneifar and Arzanpour [80] made a theoretical model for a bending-based energy harvester attached on a pneumatic inner tyre surface in which the generated electric charge was proportional to tyre speed and radial deflection. Calculation findings can be summarised to a prediction of a power generation of approximately 2.95 mW at 50 km/h when a 30 kΩ load resistor is used. Its also worth mentioning that tyre induced vibration is highly affected by road surface roughness which can change vibration velocity and acceleration amplitudes [67,81]. This can directly affect the amount of the harvested energy when a vibration energy harvester is employed.

Thermal energy is another type of energy dissipated within a pneumatic tyre structure as a result of tyre rolling, which leads to cyclic deformation in the tyre contact patch region. There are quite a few available published experimental data for tyre temperature build up under different loading and speed conditions. The following paragraph presents some published experimental and simulated pneumatic tyre temperature data under different loading and rolling speed conditions and using different techniques.

Clark and Dodge [82] presented steady state tyre pressure and temperature data; as initial tyre cavity pressure and temperature values were measured. Experiments under various loading and speed conditions were then carried out. After a period of time in which the measured values approached equilibrium, tyre air cavity pressure and temperature values were measured again. Gusakov [83] reported tyre air pressure and temperature data measured over time which were collected using a micro computer attached to the rim, from which data was downloaded subsequently. Wilburn [84] collected steady state tyre structure temperatures, e.g., sidewall and tread, using a thermal imaging technique. This technique does not actually apply to measuring air temperature inside the tyre cavity.

Due to the difficulties and challenges associated with real-time tyre condition monitoring, FEA simulation has been used by several researchers to estimate tyre structure temperature [85–88]. However, tyres are made of several composite materials which change noticeably with tyre boundary conditions. As such, simulation results remain very approximate.

The tyre rolling mechanism dictates cyclic deformation of tyres. Due to rubber mechanical properties and hysteresis loss, heat is generated in the tyres. The amount of heat generated is related to factors such as: the applied radial load and speed. On the other hand, tyre equilibrium temperature depends on, in addition to radial load and speed, other environmental conditions, such as road temperature, air flow speed, ambient temperature, the temperature of the parts surrounding the tyre, solar radiation and the distance travelled from the cold start. Tyre tread thickness, ply number and orientation, and the amount of torque applied to the wheel are also important factors in tyre cavity equilibrium temperature. Having such various environmental conditions and different tyre geometries, properties and loading conditions, it is very difficult to estimate air temperature inside the tyre cavity by using numerical methods. This raises the need for acquiring real time tyre cavity air temperature. Tyre cavity temperature could be increased by 35 °C with respect to ambient temperature in average urban driving conditions [82] and tyre tread temperature is higher than the sidewall temperature by an average of 6 °C [84]. Numerical analysis shows tyre tread temperature can rise to as high as 100 °C or more [85].

From the above, it can be seen that there is a potential source of thermal energy within the tyre structure for energy harvesting purposes, although tyre response to change in temperature is not quick enough for the TCMS application. However, the nature of a thermal energy harvesting system requires large size devices and a reasonable temperature difference which is not the case inside a rolling tyre environment. A possible example of a commercially available thermoelectric energy harvester that is utilizable for powering TPMS is the thermoelectric module (CP10,31,05) of Laird Technologies plc (London, UK) [6].

Another type of energy available inside a rolling tyre is air flow. D. Wang *et al* [55] suggested employing an air flow based piezoelectric energy harvester into the tyre cavity to power tyre pressure monitoring systems. The authors claim harvesting a maximum power of 1.1 mW with an average air flow speed of 1.083 m/s. The device however has large dimensions −758.5 mm- which make the system not very practical for a tyre monitoring system application.

In this research, direct strain energy harvesting in particular is going to be investigated and closely examined for its high power density, reasonable cost, and lowest overall mass, making it the best candidate among above described energy harvesting means for powering TPMS.

## 6. Main Methods of Energy Harvesting

The following sections review and discuss some main methods of energy harvesting in order to decide which technique is the most suited for the tyre monitoring system.

### 6.1. Electromagnetic Energy Harvesting

**Figure 6.**
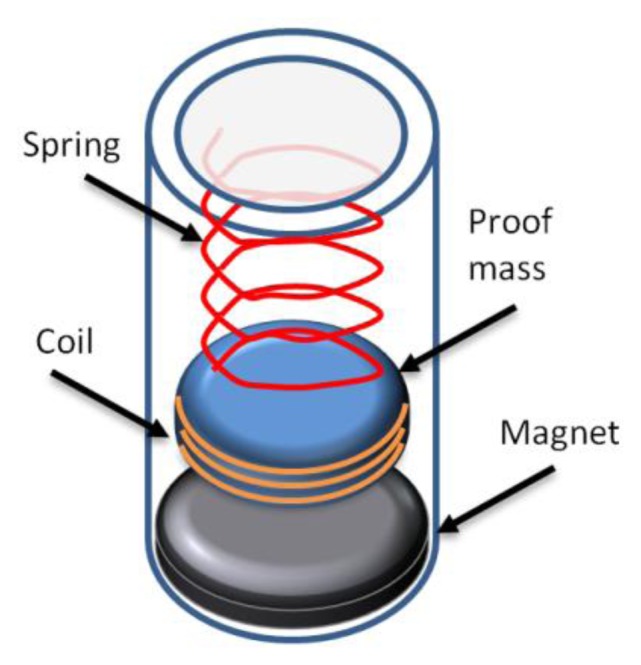
Schematic of a traditional electromagnetic energy harvester [92].

Electromagnetic, or as it is sometimes called electrodynamic, energy harvesting is based on Faraday's law of electromagnetic induction; as such, a combination of a coil and a magnet with a relative velocity between them comprises electromagnetic harvesters. To achieve this mechanism, linear vibration is primarily the most common configuration of electromagnetic energy harvesters. A typical example of a traditional electromagnetic energy harvester is shown in Figure 6 below. However, a large proportion of the harvesters in the literature have the configuration of a cantilever beam with a seismic mass attached to the free end of the beam, which can be either the magnet or the coil of the harvester [50,89]. The theory behind electromagnetic energy harvesting is explained by Saha *et al* [90]. The advantages of this type of energy harvester are its design simplicity and that it is possible to make it in such a way that its resonance frequency matches the excitation frequency for which the harvester is designed, purely by selecting the appropriate spring element. This however means that the designed system is limited to a particular frequency and only practical for constant vibration frequency applications. In addition as the harvester power generation depends on the relative velocity and change in magnetic flux, of which its amplitude is not limited by its fatigue strength, for example, piezoelectric material [91].

One of the main disadvantages of electromagnetic energy harvesters is their low output voltage and low power density [49,50,93,94], which make it difficult to harvest energy effectively, particularly for the research target application. Another fact was observed in the literature; scaling down an electromagnetic harvester to MEMS scale usually influences the system to have a resonance frequency higher than the research application frequency band width, 0 to 100 Hz [89,95]. Some of the main suppliers of commercial vibration based electromagnetic energy harvesters are Ferro Solutions Inc. (USA), MicroStrain Inc. (USA), EnOcean GmbH (Germany), and Perpetuum Ltd. (UK). A presented list for such harvesters can be found in [50,52,96].

### 6.2. Electrostatic Energy Harvesting

**Figure 7.**
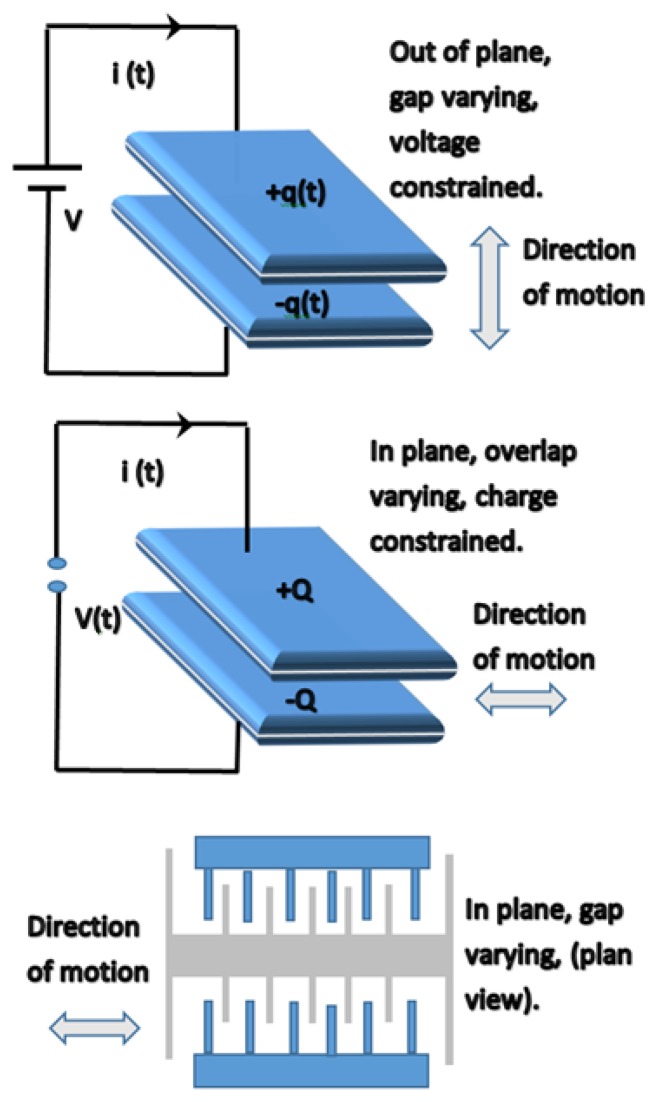
Types of electrostatic energy harvesters [93].

The electrostatic principle is the simplest among the energy harvesting techniques. It relies on the relative motion between the two plates of a charged capacitor, acting as a variable capacitor (*C*_v_), while containing the same electric charge (*Q*). Consequently, a change in the electric potential (*V*) across the capacitor will occur (*Q* = *CV*). Figure 7 below shows different types of electrostatic energy harvester configurations.

The main advantage of this type of energy harvester is their compatibility of being micromachined, integrated and produced along with application microchips, such as sensors, to act as their power source and supply them with electrical power whenever energy harvesting occurs. When energy harvesting is in the micro scale, micromachined electrostatic harvesting systems have a better coupling coefficient when compared to electromagnetic systems. In terms of the level of the direct generated voltage in electrostatic harvesters, it is usually within an appropriate level for most electronic circuitries [49]. However, there is a risk of contact between the electrodes of the capacitor [94]. Also, the fact that electrostatic harvesters have to be charged initially, or at least once in a while, due to electric charge leakage, makes them incapable of being the main power source; therefore they are more suited for recharging batteries and acting as an auxiliary power supply, which is not preferred for the research application. Design and analysis of an electrostatic energy harvester is explained in [49,93]. A recent summary of the state of the art electrostatic energy harvesters can be found in [50,96].

### 6.3. Piezoelectric Energy Harvesting

**Figure 8.**
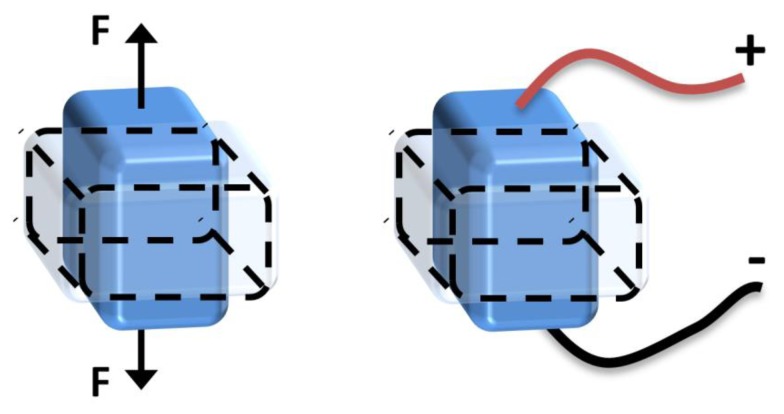
Macroscopic piezoelectric effect, direct (left), converse (right) [98].

After the discovery of the piezoelectric phenomena within certain types of materials in the late 19th century [97], the piezoelectric effect has been applied in different sensing and transduction applications. The main benefit of this effect is the direct conversion of mechanical stress, strain, or deformation, occurring in a piezoelectric material, into electrical charge and vice versa. The former is termed as the direct piezoelectric effect, which is of interest in this research, and the latter termed as the piezoelectric converse effect (Figure 8). The amount of the produced electrical charge depends on the piezoelectric material properties and in proportion with the mechanical stress or deformation applied onto it. Different mechanisms of energy harvesters based on the piezoelectric effect have been produced. A cantilever-based resonant structure is the most common among piezoelectric energy harvesters in the literature, in which mechanical vibration energy is converted to electric charge. This is probably because of the simplicity of fabricating such a design, whether in macro or micro size.

As mentioned earlier, the only issue with resonance based energy harvesters is their low performance whenever the excitation frequency is not close to the resonance frequency. However some multi-resonance structures have been promoted [99–101]. This however leads to a large harvester volume and problems associated with the harvester structure reliability [93]. Some examples of main suppliers of commercial piezoelectric vibration based energy harvesters are MicroStrain Inc. (Willston, VT, USA), EoPLEX Technologies Inc. (San Joes, CA, USA), and Mide Technology Corporation (Meddord, MA, USA). Some leading manufacturers of piezoelectric transducers for the purpose of energy harvesting are PI (Physik Instrumente) Ltd., (Karlsruhe, Germany), Smart Materials Corp., (Dresden, Germany), and Advanced Cerametrics Inc., (Lambertville, NJ, USA). The latter has been producing a product made with piezoelectric fibres, so called Piezo Fibre Composite (PFC) (Advanced Cerametrics, online [Available: http://www.advancedcerametrics.com/] [Accessed on 1 October 2010]) [102] with high mechanical to electrical energy conversion and it also has a quite light (2 g) and flexible format compared to other commercial bulk PZT elements [103].

Having the generated electric charge in a piezoelectric element proportional to the amount of subjected stress, some designs are based on harvesting the strain energy directly without the complications of a vibration mechanism [104,105]. This makes the device functional over a wider frequency range and generates energy almost linearly with the applied frequency. Coupling piezoelectric with the electromagnetic techniques is also another way to enhance the amount of harvested energy [106–108], but again it means more mass and not particularly higher power density. Different types of techniques used in piezoelectric energy harvesters are presented in [50,52,93,109].

**Figure 9.**
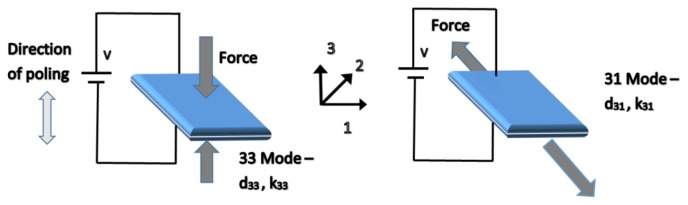
Piezoelectric constants in typical energy-harvesting modes [93].

**Figure 10.**
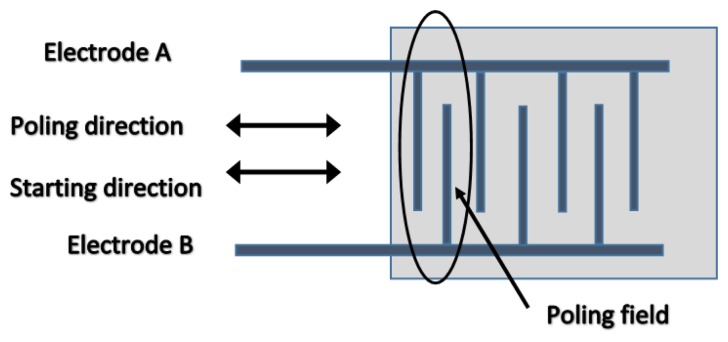
Interdigital electrode arrangement [107].

Piezoelectric harvesters can operate in two main different modes; 31 and 33 as shown in Figure 9. The vast majority of the piezoelectric energy harvester designs operate in the lateral 31 mode, particularly when the piezoelectric element is adhered onto a geometry that converts vertical displacement into a lateral strain. Some designs, however, operate on the 33 mode, which is a function of the compressive strain, because it usually has constants higher than the 31 modes. Mode 33 can be exploited, even under lateral strain loading conditions, when the piezoelectric element is structured within inter digital transducer geometry as shown in Figure 10 [93].

One of the limitations when a piezoelectric element is applied onto a flexible surface to harvest direct strain energy is the amount of strain transferred to it. This strain must not exceed a certain limit to avoid any fatigue damage in the piezoelectric element. In most commercial piezoelectric elements, the substrate -on which the piezoelectric material is printed- is brittle and has relatively low strain limits. In Princeton University, a group of researchers developed a method to print piezoelectric ceramics onto a rubber substrate to obtain a stretchable energy harvester, e.g., in implantable or wearable energy harvesting systems [110]. Further investigation is yet needed to integrate this type of energy harvester with an electronic circuit and power management units.

### 6.4. Thermal Energy Harvesting

**Figure 11.**
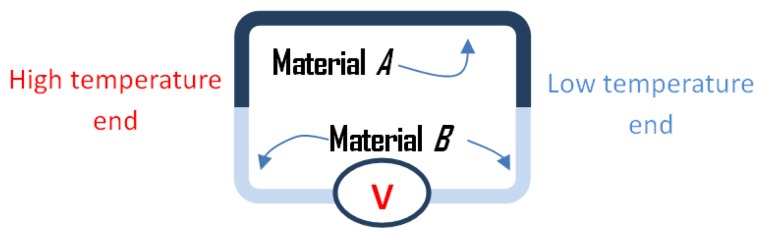
The Seebeck effect: a voltage generated by the temperature difference across the junctions [111].

Certain combinations of materials, metal alloys or some semiconductors can generate electricity when subjected to a temperature gradient [111]. This phenomenon, which is known as the Seebeck effect, has been applied in various devices in order to achieve different purposes; e.g., thermocouples for temperature measurement, and thermal energy harvesters. A schematic diagram for the Seebeck effect and a general thermal energy harvester is shown in Figure 11.

Although it is still not mature enough and more research is necessary to promote this technique, commercial thermoelectric products for thermal energy harvesting purposes are available in the market [53]. However, these harvesters are usually quite expensive. In addition, in order to generate a sustainable electric power, a considerable temperature difference is required and the average size and weight of such harvesters exceed that of the harvesters which use the techniques described earlier.

Commercially available thermoelectric devices are bulky and rigid [111], and they are not suitable to install in moving parts such as pneumatic tyres, which is the target application of this research. Some of the main suppliers of such devices are Marlow Industries Inc. (Dalas, TX, USA), Micropelt (Germany), Enocean GmbH (Munich, Germany), Nextreme Thermal Solutions (Durham, NC, USA), Tellurex Corp. (Traverse city, MI, USA), and Laird Technologies / Thermal Solutions (London, UK). The latter has developed a practical and compact design for thermal energy harvesting purposes, but its cost is still relatively high for the research target application.

Knight and Davidson [56] presented a thermal energy harvester which can generate a reasonable electrical power, sufficient for most wireless sensor nodes' applications; 50 mW when the temperature difference is 20 °C. The generated power in the latter is proportional to the temperature difference, and the device uses a relatively large space to operate efficiently [50]. Successful attempts to harvest thermal energy using micro scale thermoelectric elements for powering a wrist watch, are reported in [111], the high cost, however, makes such systems uncompetitive for commercialization. More details about thermal energy harvesters' theory and principles can be found in [111].

### 6.5. Justification of Focusing on Piezoelectric Energy Harvesting

S. Roundy is a well know researcher in the field of energy harvesting. He developed an admirable piece of work in the field of vibration based energy harvesting, including the three main methods of energy harvesting; electrostatic, piezoelectric and electromagnetic, with a detailed comparison to determine which method is the most efficient in terms of energy density [49]. He concluded that using piezoelectric offers the highest power density. Emma L. Worthington [50] carried out a comprehensive study on energy harvesting means, and made a detailed comparison of them, from which she made a similar conclusion. Similar comparisons and conclusions can be found in [51,52,93,96,112]. M. Pereyma [113] and P. Mitcheson *et al* [114] argue that at low frequency levels piezoelectric energy harvesters are more powerful than electromagnetic, however, at high frequency applications electromagnetic harvesters are more reliable. Having low frequencies with the highest extractible energy levels experienced by tyres under rolling conditions, low frequency levels piezoelectric energy extraction is more suited for the TPMS application.

One of the main advantages of the piezoelectric energy harvesting technique over the electrostatic technique is the lack of necessity for any external power source or any initial charge applied to it, which makes it a an excellent choice for the research application. The performance of each piezoelectric material depends on its material properties. The constitutive equations that relate the mechanical and electrical properties of the piezoelectric transducers are presented in [115].

## 7. Power Management Circuitry and Energy Storage Techniques

The generated electric charge in virtually all the energy harvesting techniques, and at least for those previously discussed, require power management circuitries, through which the harvested energy from the harvester is adapted to suit a particular application load. Firstly, rectification is required if the generated energy is in an alternating current, such as in electromagnetic, electrostatic and piezoelectric energy harvesting techniques. This is usually done by using a diode bridge rectifier. Then, an energy storage element is required to act as a reservoir, which can be either a rechargeable battery or a capacitor. The next stage is conditioning the output power to an appropriate voltage to supply sufficient current, depending on the nature of the system load. This can be carried out in several different ways; e.g., by using a DC-DC converter [49], or by using op-amps and comparators [116]. A voltage multiplier can be used to step-up the generated voltage especially when the voltage level of the generated charge is not high enough [117].

Various power management schemes have been introduced in order to slightly increase the efficiency of particular energy harvesters, e.g., electric tuning in which the load impedance is adjusted [93,118], impedance matching techniques [119], and a switching technique [50,120]. However, adding extra electrical components increases the cost and complexity of the system. Regarding the energy storage options, different comparisons between capacitor and rechargeable batteries are presented in the literature. Roundy [49] shows that capacitors have the advantage of the flexibility of their charging up method, and their infinite lifetime, over rechargeable batteries. In addition, rechargeable batteries' leakage escalates with the batteries' aging and becomes dominant in terms of power loss, although batteries have higher energy density than capacitors.

Guan and Liao [121] conducted experiments to investigate the efficiency of the energy element for both rechargeable batteries and super capacitors in piezoelectric based energy harvesting systems. In the former, it was found that leakage resistance is the main drawback within the energy storage element, and that super capacitors have many advantages over rechargeable batteries, e.g., leakage resistance, adaptability, lifetime, and the lack of necessity for charging protection circuitry.

H. Sodano *et al* [122] carried out experimental tests to examine different piezoelectric materials' performance in terms of their efficiencies in charging different rechargeable batteries. This showed that a piezoelectric element must be chosen carefully when it is used to recharge a particular rechargeable battery when applied in an energy harvesting system; which is not the case when a capacitor is used as the energy storage element [49].

Squires [123], from Infinite Power Solutions Inc. (Littleton, CO, USA), presented a comprehensive comparison between different energy harvesting storage devices in the first annual workshop on Micro Power Technologies in 2009. The presentation shows that high temperature (50–85 °C) is a drawback in general for all types of energy storage, and particularly for super capacitors, as it leads to reduction in their lifetime. Also, super capacitors have a high charge threshold acceptance; that is bulky energy harvesters are necessary. However, they can handle high discharge rates (>100 mA) and a high number of charge cycles (>10^6^). Rechargeable AA/AAA batteries have a high energy capacity and are suited for high power applications; however, they have high leakage rates (>40%/year), and low cycle life, 1000 at 80% depth of discharge (DOD), *i.e.,* not suited for micro power applications. Due to higher internal resistance in rechargeable coin batteries, low discharge rates (<200 μA) are a problem. Compared to AA/AAA batteries, coin batteries have lower cycle life (500–1000 at 80% DOD), although they are smaller in size and have less discharge rates (2%–5%/year), making them suitable to act as a backup power source. Thin-film batteries, the product of Infinite Power Solutions Inc. (USA), have the advantage of being very thin in geometry (25 × 25 × 0.17 mm^3^), and have a high cycle life (>10,000 at 80%DOD), low discharge rate (2%–5%/year), and very low charge acceptance threshold (<100 nA) making them well suited for micro energy harvesters. Low temperature and shelf life (<10 years), however, are dangerous enemies of energy storage. The thin-film battery product comes with its power management circuit monolithically. Other ready to use commercial power management units are available from the following suppliers; Linear Technology Corp. (Milpitas, CA, USA), MicroStrain Inc. (USA), EnOcean GmbH (Germany), Texas Instruments Inc. (Dalas, TX, USA), and Advanced Linear Devices Inc. (Sunnyvale, CA, USA).

In order to select a power management circuitry that is well suited for TPMS, essential characteristics have to be taken into consideration. For instance, it is desirable to utilize a high efficiency energy storage unit; that is by minimizing electric charge leakage and increasing reliability against tyre thermodynamics and kinetics. Another important property is system durability. Charging-discharging cycles and shelf life are expected to last long enough for to be installed within a passenger vehicle tyre. This is at least approximately a travelling distance of 20 thousand miles. As wheel balance is crucial for safe and convenient driving, system weight and volume preferred to be minimal. Energy requirement for TPMS evokes using an energy source and storage that can supply steady voltage while a relatively high current in a short time; that is pulsatile loading which occurs during signal transmission. It is worth mentioning that signal transmission drains most of harvested energy if high transmission rate is needed. Taking sensors measurements consumes the second biggest portion of harvested energy, but still less than signal transmission power consumption even if high sampling rate is used. All above indispensible characteristics and energy requirements suggest using an electric capacitor for energy storage as it can have very small leakage, adapts to pulsatile loading, and more resistive to rolling tyre thermodynamics than other energy storage schemes, e.g., batteries.

## 8. Tyre Monitoring Systems

In this section, the state-of-the-art of tyre monitoring systems is presented. The idea of mounting a tyre monitoring system first started in order to monitor tyre inflation pressure, the most crucial parameter on which tyre performance relies. The first mounted system was for a Porsche 959 in 1986 [124], and since then, millions of such devices have been installed in other cars [125]. Not only pressure measurement has been applied in tyre monitoring systems, but in addition air temperature, as the second most important parameter to measure in a rolling tyre. Other tyre parameters, e.g., strain, acceleration, wear, *etc.* are being investigated and monitoring systems to include measuring these parameters are under development [62,126] in order to promote an intelligent tyre system.

In general, tyre monitoring systems focus on the measurement of tyre inflation pressure, especially after establishing the TPMS legislation (Standard No. 138 (Lemon Law Center, online, [Available: http://lemon.onecle.com/fmvss-standard-no-138/] [Accessed on: 4 May 2012])) [15,127]. The main reasons why such legislation was created were the reported deaths (over 100) and injuries (about 700) due to rollover car accidents in the late 1990s [128].

Tyre inflation pressure, in the conventional way, can be monitored using air pressure sensors; that is a direct TPMS, or using unconventional methods when other parameters are measured; that is an indirect TPMS, e.g., by measuring tyres' angular speeds individually and comparing them to each other [129,130]. Indirect TPMSs existed as a cheaper alternative for direct TPMSs, but at the price of accuracy and detection time. Moreover, preliminary statistical and simulation data show that an indirect TPMS can only detect half of existing under-inflations by 25% or more [131], because in some cases, under-inflation is undetectable in an indirect TPMS; e.g., when two tyres are on the same side, on the same axle, or all four tyres are equally under-inflated. A compromise solution is a hybrid TPMS; that is a combination of both direct and indirect TPMSs, in which fewer tyre pressure sensors are used. In the latter only two diagonally located tyres are equipped with both direct and indirect TPMSs [28].

Direct TPMSs can be either battery powered; that is an active system, which is the case for the vast majority of the TPMS products on the market, or remotely powered; that is a passive system, thereby the system receives its required energy wirelessly, e.g., by means of an electromagnetic field [125]. This system has a major installation drawback due to the need for a complex wiring network to be installed for both powering and reading out purposes. A general comparison between direct and indirect TPMSs is shown in Table 2. A wide range of various TPMS types is presented in [132].

In the literature, various techniques have been applied in TPMS designs for powering and/or transmitting tyre inflation pressure data; e.g., RFID, SAW, BAW, and induction loop. Similarly, different mechanisms of energy harvesting methods to supply a TPMS with the required electrical energy have been investigated, and based on different types of energies; e.g., vibration , direct strain, tyre-rim assembly kinematics, bending within the tyre structure, and air flow inside the tyre. Table 3, Table 4, and Table 5 show state of the art various published TPMS designs; possibly more are being researched and investigated but are not accessible due to patent reasons.

Active TPMSs are conventionally powered by batteries. Battery-less TPMSs usually refers to remotely powered systems (passive TPMS), but also can be used to describe a TPMS powered by energy harvesting. The former has a hidden disadvantage; that is draining the vehicle battery [133], as remotely powering is actually using the vehicle battery and thereby they are technically battery powered. The latter is the type of TPMS that is legitimately battery-less, so called self-powered TPMS which is the target of this research. The SAW sensor, for example, consumes around 10 mW [134], which is still low comparing to other car electrical devices, e.g., headlights, which consume around 50 W (MK6 Ford Fiesta). However, active TPMSs power consumes less than passive TPMSs, with a power consumption around 200–250 µ Ws for a Stateof-the-Art TPMS module [135].

From Table 3, Table 4, and Table 5 it can be seen that a lot of energy harvesting approaches for powering TPMSs have been investigated, however, only a few were actually applied into a tyre-rim assembly. This is due to the challenges associated with having such a system installed within a tyre-rim assembly, particularly the extreme levels of centrifugal acceleration of up to 3000g [23].

## 9. Battery-Less TPMS Industry

Virtually all the commercially available TPMSs are battery powered and designed to be mounted on the rim or fixed to the tyre valve. However, some companies are currently working on developing a battery-free TPMS. For instance, Transense Technologies plc is using a surface acoustic wave (SAW) based technology, in addition to companies such as Honeywell (Weybridge,UK) and Michelin (Greenville, SC, USA). SAW systems measure pressure with temperature compensation and can either be attached to the wheel (e.g., back of valve or to run-flat system), embedded in the wheel or it can be attached to the tyre itself [3,139,156]. On the other hand, Visityre (East Kalara, Australia) is using an electromagnetic closed-coupling technology. In this design, relatively large hardware is needed to be installed in the tyre brake structure [3,137,157]. The Hong Kong Applied Science and Technology Research Institute Company Ltd. (ASTRI, Hong Kong) have recently developed a piezoelectric vibration-based energy harvesting TPMS. Although the specification of this device is not precisely clear, ASTRI claims that the commercial model of this device can be reduced in size to 2.5 × 2.5cm [3,158]. Eoplex is also developing a vibration energy harvester. This company focuses only on producing the energy harvester to be used in energy scavenging based TPMS systems as a power supply [3,159]. Perilli (Milan, Italy), in collaboration with Schrader Electronics Ltd. (Antrim, UK), under the Cyber Tire Lean project, has developed a tyre monitoring system, approximately 24mm in diameter with a flexible rubber base, that can be attached to the inside surface of the tyre. The system is capable of measuring pressure, temperature, the average load supported by each wheel and speed, sending the information directly to the electronic control unit of the vehicle the device harvests energy through tyre mechanical vibration and supplies the collected energy to the device circuitry [160]. Siemens VDO (Regensburg, Germany), now Continental, collaborating with Goodyear Tyre and Rubber Co. (Akron, OH, USA), has developed a TPMS that can be mounted in the tyre's rim. The device is powered by a piezoelectric based vibration energy harvester. The manufacturer claims that this device is capable of transmitting data every 60 s or less [160]. In vibration-based energy harvesting systems, there are two main inescapable problems. Firstly, these systems need to operate at a particular value of vibration frequency in order to achieve optimum performance. Otherwise, a slight difference in the vibration frequency decreases the efficiency of this vibration system dramatically. The second problem is the size and weight issues with these systems (energy density), if a reasonable amount of power generation is required [78]. Piezotag has developed a TPMS which can be mounted on the inside surface of the tyre and it harvests energy from tyre deformation, particularly bending [3,161].

## 10. Conclusions and Discussion

The literature review began with the history of pneumatic tyres and how they evolved to their current form in which the inflation pressure is a crucial factor for safety and durability. There are wireless devices that can monitor tyre inflation pressure and other conditions when necessary in real time. Then a survey of all the essential components that are needed to build a TCMS was carried out. Following this there was a review of different types of pressure sensing mechanisms, making a comparison between them, and choosing the mechanism that meets the self-powered TCMS requirements. Then the necessity to solve the energy source problem for powering tyre monitoring systems, particularly inflation pressure monitoring, was presented. Following this various harvestable energy sources in a rolling tyre were discussed, kinetic energy, strain energy, and thermal energy. Then an overview of the existing energy harvesting techniques, e.g., piezoelectric, electromagnetic, electrostatic, and thermoelectric, that can be applied within the tyre-rim assembly was given and compared, in order to choose the most appropriate technique that meets the power requirements for the TCMS and that can withstand the tyre cavity harsh conditions. Following this different means of power management techniques in order to store and condition the harvested energy were presented. Finally a survey of the existing battery-less TPMS, self-powered TPMS, and energy harvesting devices which are based on scavenging energy from a rolling tyre, were presented. The general TPMS requirements are shown in Table 6. These requirements are used as design criteria for the tyre monitoring system to be developed in this research project.

It has been found that the least power hungry pressure sensor is the capacitive type, and therefore a design incorporating this type of pressure sensor is needed in this study.

In terms of harvesting the energy required to power the TCMS, it has been found that the most efficient technique is harvesting strain energy in the tyre, which is generated by cyclic tyre deformation, directly; that is by applying a piezoelectric element onto the inner tyre liner in an appropriate position. A vibration based energy harvesting system utilizing a weighted cantilever system or an electromagnetic system could also be efficient. However the drawbacks of these systems are a narrow response frequency for useful power generation and an increase in system mass resulting in lower power density.

Experimental tyre strain measurements and profiles obtained by other studies [58,65,163,164] have been used to estimate the amount of electric charge generated when a piezoelectric material is attached to an inner tyre surface. The only limitation, however, is excessive tyre strain. This means an appropriate adhesive has to be used to keep the piezoelectric harvester functional and to prevent permanent damage.

Different types of TPMSs are reviewed in this article. Active TPMS means that the system has its own power source, which can be either a battery or an energy harvesting based power source. On the other hand, a passive TPMS is driven remotely and therefore no direct connection with its power source exists. This makes passive TPMSs not well suited for low power consumption purposes as transferring power wirelessly is not an efficient process. For this reason, active TPMS approach is targeted in this research. TPMS pressure sensing approaches are also reviewed in this article. There are three main pressure sensing methods; direct, indirect and hybrid, among which Direct TPMS offers the greatest accuracy and the quickest possible response. For these reasons, direct TPMS is targeted in this research.

The major power sources for active TPMSs are reviewed in this article. It was found from the literature that batteries are the predominant power supply for TPMS devices for their low cost and high power density. Batteries however have considerable downsides which are their limited life time and subsequently environmental disposal problem. Therefore energy harvesting is targeted in this research. High energy density and power conversion efficiency are crucial factors to power the targeted TCMS. The main energy harvesting techniques observed in the literature are electromagnetic, electrostatic, piezoelectric and thermal energy harvesting means. The output signal characteristics (voltage and current) are also required to be within a range that is possibly adaptable by the existing power management chips. The best candidate among the major energy harvesting techniques which meets the selection criteria is the piezoelectric energy harvesting technique, and therefore research is focused on this type of energy harvesters.

In the literature, it has also been found that an appropriate circuitry has to be built to maximize the collectable harvested energy efficiently, and therefore an electronic circuit for power management, data measurements, and wireless transmission purposes is needed in this study. The circuit design is driven by electronic chips available on the market today and could possibly evolve over the study period depending on any new related electronic chips which are developed.

There are several mechanisms to employ piezoelectric transducer as energy harvesters, two among which are of interest in this research; vibration and direct strain energy harvesting mechanisms. In the literature, vibration energy harvesting approach is the dominant approach for piezoelectric-based energy harvesting devices, including TPMS related research, due to its simplicity to assemble, to model, and to affix to vibration sources. Direct strain energy harvesting approach is an attractive way to harvest energy, besides it is easier to model. However the literature lacks experimental investigation for such harvesting mechanism, particularly in rolling tyres. Further exploration for vibration and direct strain energy harvesting mechanisms in a rolling tyre was found to be needed to determine which method suits powering TPMS best. For this reason, comparison between vibration and direct strain energy harvesting for powering TPMS purposes is addressed in this research.

## Figures and Tables

**Table 1.** Performance Features of Resonant, Piezoresistive, and Capacitive Sensing [39,45].

**Feature**	**Resonant**	**Piezoresistive**	**Capacitive**
Output form	Frequency	Voltage	Voltage
Resolution	1 part in 10^8^	1 part in 10^5^	1 part in 10^4^–10^5^
Accuracy	100–1000 ppm	500–10,000 ppm	100–10,000 ppm
Power consumption	0.1–10 mW	≈10 mW	<0.1 mW
Temperature cross-sensitivity	–30 × 10^−6^/°C	–1,600 × 10^−6^/°C	4 × 10^−6^/°C
Complexity	High	Low	Low

**Table 2.** Comparison between direct and indirect TPMSs [29,136].

	**Direct TPMS**	**Indirect TPMS**
Costs	High	Low
Physical parameters	Pressure and temperature	Rolling speed
Hardware	Wheel sensor, receiver and display	ABS sensor, ABS control unit, display
Measuring of pressure	Absolute	Relative (30% aberration from set value)
Measuring of temperature	Yes	No
Target values	Fixed maximum pressure is to be set	Learning of default values is required
Detection time	Almost real time, independent of driving manoeuvre, while moving or at rest	0.6 bar under-inflation are detected within 5 min at 130 km/h (e.g., Dunlop WarnAir)
Susceptibility of wheel components to damage during tyre installation and removal	More likely	Less likely
Need for an independent power supply	Yes	No
Need to reset after a vehicle's tyres are replaced or rotated	Yes	Yes, system must recalibrated too
Ability to detect loss of air if all tyres lose pressure	Yes	No
Ability to detect small pressure losses	Yes	No
Ability to detect under-inflated tyre while vehicle is stationary	Yes	No, vehicle must be moving
Ability to detect which tyre is underinflated	Yes	No
Susceptibility to giving false indications of a significantly under-inflated tyre	No	Yes, if the vehicle is being driven on gravel or bumpy roads at high speeds (112 km/h) or if it has mismatched tyres of a tyre out balance or out of alignment

**Table 3.** Summary of published research for various types of battery powered TPMS for passenger vehicles.

**Power Source**	**Size**	**Pressure Sensor Type**	**Other sensors within the System**	**Location within the Tyre**	**Source**
Lithium battery	-	A magnet and Hall effect IC	-	On wheel disk	Uemura *et al.,* 1985 [129]
Lithium battery	1.2 × 1.3 × 0.64 cm^3^	BAW	temperature	Not tested, expected to be applied on tyre inner liner	Flatscher *et al.,* 2009 [23]
Lithium battery	-	Capacitive	temperature	-	Rudolf *et al.,* 1997 [22]
Lithium battery	-	Piezoresistive	temperature	Rim mounted	Jian Zhang *et al.,* 2011 [24]
Passive (Magnetic coupling)	-	Piezoresistive	temperature, force vector	Rim and hub	VisiTyre, 2003 [137]
Passive	-	-	-	-	Kurashige *et al.,* 1982 [138]
Passive (SAW)	-	SAW	temperature	Valve stem	Transense, 2006 [139]
Passive (SAW)	-	Hybrid (SAW and capacitive)	temperature	Rim mounted	Siemens, 2000 [140]
Vehicle battery	-	(Indirect) wheel velocity	-	-	Qi Zhang *et al.,* 2009 [130]
Passive (RFID)	-	-	temperature	Annular, attached to inner tyre liner	Deicke *et al.,* 2009 [141]
Passive (magnetic coupling)	-	Capacitive using LC circuit	temperature	Proposed design	Nabipoor and Majlis, 2006 [142]
Passive (RFID)	-	Capacitive (MPXY8000)	temperature	Rim mounted (prototype)	Ho *et al.,* 2009 [143]
Passive (RFID) +Electromagnetic energy harvesting	-	BAW	temperature	Dedicated design, mounted on inner tyre liner	Gruber *et al.,* 2010 [144]
Power source	Size	Pressure sensor type	Other sensors within the system	Location within the tyre	Source
Passive (Magnetic coupling)	Diameter = 2.5 cm × 1 cm + coupling ring (antenna)	BAW (cantilever -based quartz resonator linked to a pressure sensitive diaphragm)	-	Fixed at the rim of the wheel	Grossmann, 1999 [145]
Passive (RFID)	-	BAW (quartz-based resonator with acoustic mirror)	-	-	Flatscher *et al,* 2008 [146]
Passive (SAW)	7 × 5.3 mm^2^ (approximately)	SAW	-	Built inside the tyre valve housing	Varadan *et al,* 2001 [147]

**Table 4.** Summary of published research for self-powered TPMS using various energy harvesting means.

**Energy Harvesting Mechanism**	**Harvesting Technique**	**Size**	**Pressure Sensor Type**	**Other Sensors within the System**	**Location within the Tyre**	**Source**
Compression of a very thin brass reinforced PZT at the tyre-bead rim interface	Piezoelectric (the amount of the harvested energy is directly proportional with tyre speed)	-	Piezoresistive pressure sensor (CPX100G)	-	Mounted at the tyre-bead rim interface	Makki and Pop-Iliev, March 2012 [148,149]
Bending	Piezoelectric (the amount of the harvested energy is directly proportional with tyre speed)	-	Piezoresistive pressure sensor (CPX100G)	-	Attached onto the inner surface of the tyre belt	Makki and Pop-Iliev, 2011 [59,150]
Cantilever beam vibration (oscillates in the tangential direction)	Piezoelectric (the amount of the harvested energy is inversely proportional with tyre speed)	Contained in a traditional TPMS casing (40–60 × 25–30 × 8–10) mm^3^	LV Sensors' *i*TPS™	two-axis accelerometer	Fixed to the tyre valve-stem and sits on the rim	Atmel Corporation, 2008 [20,151]

**Table 5.** Summary of published research for energy harvesters designed potentially for self-powered TPMS.

**Energy Harvesting Mechanism**	**Harvesting Technique**		**Size**	**Power Output**	**Output Voltage**	**Location within the Tyre**	**Source**
Vibration (floating magnet oscillates inside cylindrical coil)	Electromagnetic (the amount of the harvested energy is directly proportional with tyre speed)		5 mm diameter × 5 mm high magnet, no more specifications are given	0.054 mW at 60 km/h (by using a shaker to simulate the harvested energy, tyre size is not specified)	1.5 VAC at 60 km/h	Attached onto the inner surface of the tyre belt	Tornincasa *et al,* 2012 [79]
Vibration (Piezoelectric Bender Generator)		Piezoelectric (the harvested energy is directly proportional with tyre speed)	31.8 × 3.2 × 0.66 mm^3^	0.78 μW at 50 km/h 2.99 μW at 80 km/h	2–3 V at 50 km/h 5-10 V at 80 km/h	Attached to the tyre wall from the outside in the tangential direction at 16 cm distance from the wheel centre	Pinna, 2010 [152]
Vibration (Piezoelectric cantilever with a seismic mass)		Piezoelectric (the amount of the harvested power peaks at the resonance frequency)	55.4 × 15.2 × 1.2 mm^3^	100.4 μW at resonance frequency (47.6 Hz)	6 VAC at resonance frequency (47.6 Hz)	-	Chen and Pan, 2011 [77]
Vibration (Piezoelectric MEMS cantilever oscillates in the radial direction - pulse excitation- one pulse per one tyre revolution)		Piezoelectric (the harvested energy is directly proportional with tyre speed)	Some 10 mm^2^ in area x 80 μm in thickness	5.5 μW at resonance (11 kHz)	3.7 VAC at resonance (11 kHzs)	Either on the rim or in the inner liner of the tyre	Frey, 2011 [153]
Bending (A cantilever bends in the tangential direction -pulse excitation- two pulses per one tyre revolution)		Piezoelectric (Nano-generators were integrated onto a tyre's inner surface, the amount of the harvested energy is directly proportional with the tyre speed)	1.5 × 0.5 cm2 (thickness is not specified)	peak power of 1.5 Vp × 25 nA (The deformation of the tyre during the rotation was simulated by squeezing the tyre periodically; the travel distance of the board was 12 mm with an acceleration of 30 m s-2)	1.5 Vp (same testing conditions)	attached tightly on the inner surface of the tyre using adhesive tape	Hu *et al,* 2011 [154]
Vibration (oscillation occurs in the tangential direction of the tyre)		Electromagnetic (A stacked magnet oscillates along the axis of a pick-up coil, the amount of the harvested energy varies with tyre speed, however the harvester resonates at 46 Hz)	30 × 30 × 11.7 mm^3^	0.144 mW at 50 km/h at an acceleration of 6 g, 0.4 mW at 150 km/h at an acceleration of 15g (by an arbitrary signal generator to simulate the harvested energy; 1 Hz = 6.57 km/h, that is for a 57 cm tyre diameter)	120 mV at 50 km/h at an acceleration of 6 g, 200 mV at 150 km/h at an acceleration of 15g (across 100 Ω resister)	-	Hatipoglu and Urey, 2009 [75]
Vibration (oscillation occurs in the radial direction of the tyre)		Piezoelectric (a both-side supported beam with non-linear spring stiffness central deflection)	A diameter of 10.4 mm × 1.4 mm thickness	Peak power = 80 μW at 80 km/h, average power of 40 μW over 30–180 km/h speed range	Maximum of 40 V (open circuit) conditions under which this voltage is obtained is not specified	The sensor module mounted at the inner tread area	Keck, 2007 [74]
Vibration (cantilever beam structure)	Piezoelectric (three piezoceramics in parallel connection)		Not specified	Averaged power of 0.38 mW (3 V regulated voltage and current pulses of 9.7 mA which lasts for 19.2 ms per 1423 ms across a resistor load of 3000 Ω)	Maximum 12.3 V at resonance, 125.8 Hz (open circuit)	-	Liji Wu *et al,* 2009 [69]
Vibration (oscillation occurs in the radial direction)	Piezoelectric (Air-spaced cantilever, the amount of the harvested power peaks at the resonance frequency)		15 × 6 × 46 mm^3^	47 μW at approximately 80 km/h (resonance)	>5 VAC but not specified	The vibration energy harvesting device was mounted on the wheel up-side-down to make sure the PZT operates in compression mode	Zheng *et al,* 2009 [70]
Vibration (pendulum-driven self-tuning energy harvester oscillates in the tyre tangential direction)	Piezoelectric (Pendulum impact harvester in which its ball impacts the PZT beam, the amount of the harvested energy is directly proportional with the angular speed)		(≈10) × 20 × 20 mm^3^	123 μW at 16.2 Hz 60 μW at 6.2 Hz	(21–25) V_p-p_ over the frequency range (4–16) Hz in which the system almost remains at resonance	The device is mounted at optimal radius of 7.5 mm from the centre of rotation	Lei Gu, and Livermore, 2010 [155]
Vibration (non-contact frequency up-conversion piezoelectric harvester based on magnetic repulsive force oscillates in the tyre tangential direction)	Piezoelectric (2 cantilevers excited by magnetic repulsive force)		Not clear	Average power of 10 μW over the frequency range (10–22) Hz under 1g acceleration of excitation	Average voltage ≈ 14V_p-p_ across a 6.1 MΩ resistive load	The harvester mounted on the rim inside the tyre cavity	Tang *et al,* 2012 [72]
Vibration (oscillation occurs in the tyre tangential direction)	Piezoelectric (Thunder™ piezoelectric generator)		2 cm^3^	4 mW at 800 rpm (by using a 0.12 m diameter test wheel; equivalent to a vehicle wheel with a diameter of 13 inches and a linear speed of 45.44 km/h)	Pulses of up to 150 V_0-p_	To be mounted on the rim (the energy harvester was kept in horizontal position while wheel rotation is in a vertical plane	Manl *et al,* 2009 [16]
Vibration (a weighted-pendulum which oscillate in the rotation plane)	Electromagnetic (eight permanent magnets in a disk shape, allocated in a ring, and eight corresponding coils in series are fixed on the wheel)		≈ 2.5 cm in diameter, thickness is not specified	Average power of 349 μW at 400 rpm across a 330 Ω resistive load	≈ 0.33 V_rms_ at 400 rpm across a 330 Ω resistive load	The weighted pendulum combined with magnets and coils was mounted on a rotation plate driven by an AC servo motor to simulate the device oscillation.	Wang *et al,* 2012 [73]

**Table 6.** TCMS requirements and design criteria.

**TPMS Type**	**Active and Direct**
Power supply	Energy harvesting technique
Energy harvester	To operate efficiently and to generate sufficient energy to power the TCMS. The harvester is intended to last the lifetime of the tyre (20 k–40 k miles)
Operating temperature	-40°C to 125 °C
Pressure monitoring range	0 to 3 bars (0 to 300 kPa)
Operating frequency	2.4 GHz
Pressure reading accuracy	±7 kPa (±1 psi)
Temperature reading accuracy	±1 °C
Transmission power	0-5 dBm
Voltage	3.6 V
Power consumption	Less than 250 μW
Duty cycle	To report faulty tyre pressure (reduction of up to 20% of inflation pressure) after no more than 10 minutes from detecting it and to transmit tyre pressure reading within not more than 60 minutes of cumulative driving (by summing up time while vehicle speed is larger than 40 km/h) [162]
Sensor weight	Less than 35 g
Reception unit	USB unit plugged to a portable laptop
Installation	To be affixed to the tyre valve stem inside the tyre cavity

## References

[1] TNO, IEEP, a. LAT. (2006). Review and Analysis of the Reduction Potential and Costs of Technological and other Measures to Reduce CO_2_-emissions from Passenger Cars.

[2] Transense SAW Based Tyre Pressure Monitoring System. http://www.transense.co.uk/technologies/temppressure/motorsport-tpms.

[3] Zervos H. Tyre pressure monitoring systems and energy harvesting. http://www.energyharvestingjournal.com/articles/tyre-pressure-monitoring-systems-and-energy-harvesting-00002587.asp?sessionid=1.

[4] Spectrum Batteries Inc. Lithium/Thionyl Chloride Batteries–180C. http://www.etv.com.au/Thionyl180msds.pdf.

[5] VisiTyre Automotive PL The Problems with Batteries in TPMS Safety and Environmental Concerns, in DMS 02062009 Battery Environmental Concerns. http://www.etv.com.au/Safety_Environmental.pdf.

[6] Delta Electronics Inc. Electromagnetic coupling. http://www.delta.com.tw/product/ap/tpms/tpms_main.asp.

[7] David S., Alex B., Gaby L., Pedder S. (2011). Trends in the Global Tire Industry.

[8] (2000). Title 49 United States Code 30101, Transportation Recall Enhancement, Accountability, and Documentation (TREAD) Act, Volume: Public Law 106–414-NOV.1.

[9] Smith J.A., Moore J.S., Holmbraker S., Bartlett A.J., Campbell W.A. (2000). Tiresafe Product Overview; Volume: NHTSA-00–8572–1.

[10] Mazzae E.N., Ranney T.A. (2001). Development of an Automotive Icon for Indication of Significant Tire Underinflation. Proc. Human Factors Ergonom. Soc. Annual Meet..

[11] Grygier P., Garrott W.R., Mazzae E.N., MacIssac J.D., Hoover R.L., Elsasser D., Ranney T.A. (2001). An evaluation of existing tire pressure monitoring systems; Volume: DOT 809 297.

[12] MacIsacc J.D.J., Garott W.R. (2002). Preliminary findings of the effect of tire inflation pressure on the peak and slide coefficients of friction; Volume:DOT 809428.

[13] (2000). Federal motor vehicle safety standards; tire pressure monitoring systems; controls and displays; Volume: NHTSA-2000–8572.

[14] (2005). Federal motor vehicle safety standards; tire pressure monitoring systems; controls and displays; Volume: NHTSA-2005–20586.

[15] Fleming B. (2009). Tire pressure-monitoring systems rollout (Automotive Electronics). IEEE Veh. Technol. Mag..

[16] Manla G., White N.M., Tudor J. Harvesting energy from vehicle wheels.

[17] Onoda T. (2009). IEA policies-G8 recommendations and an afterwards. Energy Policy.

[18] Rui X.H., Jin Y., Feng X.Y., Zhang L.C., Chen C.H. (2010). A comparative study on the low-temperature performance of LiFePO_4_/C and Li_3_V_2_(PO_4_)^3^/C cathodes for lithium-ion batteries. J. Power Sources.

[19] Zhang S.S., Xu K., Jow T.R. (2003). The low temperature performance of Li-ion batteries. J. Power Sources.

[20] Roundy S. Energy Harvesting for Tire Pressure Monitoring Systems, Design Considerations.

[21] Löhndorf M., KvisterØy T., Westby E., Halvorsen E. Evaluation of Energy Harvesting Concepts for Tire Pressure Monitoring Systems.

[22] Rudolf S.S.F., Hoogerwerf A. Components for battery-powered wireless tire pressure and temperature monitoring systems.

[23] Flatscher M., Dielacher M., Herndl T., Lentsch T., Matischek R., Prainsack J., Pribyl W., Theuss H., Weber W. A robust wireless sensor node for in-tire-pressure monitoring.

[24] Zhang J., Chen T., Kong X.M., Ren T.L., Liu L.T. (2011). A Tire Pressure Monitoring System Based on MEMS Sensor. Key Eng. Mater..

[25] Tompkins E.P. (1981). Dunlop Archive the History of the Pneumatic Tyre.

[26] Gillespie T.D. (1992). Fundamentals of Vehicle Dynamics.

[27] Thurau J., Ruohio J., Jürgen V., Gessner W. (2004). Silicon Capacitive Absolute Pressure Sensor Elements for Battery-less and Low Power Tire Pressure Monitoring. Advanced Microsystems Automotive Applications.

[28] Velupillai S., Guvenc L. (2007). Tire Pressure Monitoring (Applications of Control). IEEE Contr. Syst..

[29] Walter R., Salzinger T. (2003). Final report, Motor vehicle tyres and related aspects, TÜV Automotive GmbH.

[30] Steckelmacher W. (1965). A review of low pressure measurement from an industrial viewpoint. J. Sci. Instrum..

[31] Strangeways I. (2002). Back to basics: The “met. enclosure”: Part 8(b) –Barometric pressure, aneroid barometers. Weather.

[32] Hunt L.B. (1944). The History of Pressure Responsive Elements. J. Sci. Instrum..

[33] Tang Y.U. (2007). Michigan State, Polycrystalline diamond (poly-C) technology and piezoresistive sensor application for cochlear prosthesis.

[34] Busch-Vishniac I.J. (1999). Electromechanical Sensors and Actuators.

[35] Eaton W.P., Smith J.H. (1997). Micromachined pressure sensors: Review and recent developments. Smart Mater. Struct..

[36] Anand K.B., Kulkarni V.A. (2009). Metrology & Measurement.

[37] Eddy D.S., Sparks D.R. (1998). Application of MEMS technology in automotive sensors and actuators. Proc. IEEE.

[38] Barlian A.A., Park W.T., Mallon J.R., Rastegar A.J., Pruitt B.L. (2009). Review: Semiconductor Piezoresistance for Microsystems. Proc. IEEE.

[39] Beeby S., Ensell G., Kraft M., White N. (2004). MEMS Mechanical Sensors.

[40] Gad-el-Hak M. (2002). The MEMS Handbook.

[41] Madhavi K.Y., Sumithradevi K.A., Krishna M., Vijayalakshmi M.N. Analysis of Square and Circular Diaphragms for a MEMS Pressure Sensor Using a Data Mining Tool.

[42] Khakpour R., Mansouri S., Bahadorimehr A.R. Analytical comparison for square, rectangular and circular diaphragms in MEMS applications.

[43] Hermann S. (1991). Low-pressure sensor with bossed diaphragm. Mechatronics.

[44] Cavalloni C., Berg J., Krueger S., Gessner W. (2002). Overview: Principles and Technologies for Pressure Sensors for Automotive Applications. Advanced Microsystems for Automotive Applications Yearbook 2002.

[45] Kinnell P.K. (2005). The development of selective strain coupling structures for a novel MEMS resonant pressure sensor. Ph.D. Thesis.

[46] Wang Q., Ko W.H. (1999). Modeling of touch mode capacitive sensors and diaphragms. Sens. Actuators A Phys..

[47] Wei-Zang C., Li-zhou P., Xiao-ming L. (1992). Large deflection problem of a clamped elliptical plate subjected to uniform pressure. Appl. Mathem. Mech..

[48] Timoshenko S. (1940). Theory of Plates and Shells.

[49] Roundy S.J. (2003). Energy Scavenging for Wireless Sensor Nodes with a Focus on Vibration to Electricity Conversion. Ph.D. Thesis.

[50] Worthington E.L. (2010). Piezoelectric Energy Harvesting: Enhancing Power Output by Device Optimisation and Circuit Techniques, in School of Applied Sciences Microsystems and Nanotechnology Centre. Ph.D. Thesis.

[51] Chalasani S., Conrad J.M. A survey of energy harvesting sources for embedded systems.

[52] Cook-Chennault K.A., Sastry A.M., Thambi N. (2008). Topical review: Powering MEMS portable devices–a review of non-regenerative and regenerative power supply systems with special emphasis on piezoelectric energy harvesting systems.

[53] Knight C., Davidson J., Behrens S.B. (2008). Energy Options for Wireless Sensor Nodes. Sensors.

[54] Knight C., Davidson J., Mukhopadhyay S.C., Leung H. (2010). Thermal Energy Harvesting for Wireless Sensor Nodes with Case Studies. Advances in Wireless Sensors and Sensor Networks.

[55] Wang D.A., Pham H.T., Chao C.W., Chen J.M. A Piezoelectric Energy Harvester Based on Pressure Fluctuations in Kármán Vortex Street.

[56] Knight C., Davidson J. Thermoelectric energy harvesting as a wireless sensor node power source.

[57] Ende D.A.V.D., Wiel H.J.V.D., Groen W.A., Zwaag S.V.D. (2011). Direct strain energy harvesting in automobile tires using piezoelectric PZT–polymer composites. Smart Mater. Struct..

[58] APOLLO (2005). Intelligent Tyre for Accident–Free Traffic.

[59] Makki N. Piezoelectric power generation in automotive tires.

[60] Jingang Y. A. (2008). Piezo-Sensor-Based “Smart Tire” System for Mobile Robots and Vehicles. IEEE/ASME Trans. Mechatr..

[61] Holtschulze J., Goertz H., semann T. (2005). “A simplified tyre model for intelligent tyres,” Vehicle System Dynamics.

[62] Ryosuke M., Naoki H., Todoroki A., Mizutani Y. (2010). Analysis of Applied Load Estimation Using Strain for Intelligent Tires. J. Solid Mech. Mater. Eng..

[63] Hall J.T.M.W., Jones R.P. (2004). Finite element simulation of a rolling automobile tyre to understand its transient macroscopic behavior. J. Automob. Eng..

[64] Pottinger M.G. (1992). The three dimensional contact patch stress field of solid and pneumatic tires. Tire Sci. Technol..

[65] Yang X. (2011). Finite Element Analysis and Experimental Investigation of Tyre Characteristics for Developing Strain-Based Intelligent Tyre System, in Mechanical Engineering. Ph.D. Thesis.

[66] Gardner J.D., Queise B.J. (2006). Introduction to Tire Safety, Durability and Failure Analysis. The Pneumatic Tire.

[67] Braghin F., Brusarosco M., Cheli F., Cigada A., Manzoni S., Mancosu F. (2006). Measurement of contact forces and patch features by means of accelerometers fixed inside the tire to improve future car active control. Veh. Syst. Dyn..

[68] Kindt P., Sas P., Desmet W. (2009). Measurement and analysis of rolling tire vibrations. Opt. Lasers Eng..

[69] Liji W., Yixiang W., Chen J., Chun Z. Battery-less piezoceramics mode energy harvesting for automobile TPMS.

[70] Zheng Q., Tu H., Agee A., Xu Y. Vibration Energy Harvesting Device Based on Asymmetric Air-Spaced Cantilevers for Tire Pressure Monitoring System.

[71] Gu L., Livermore C. (2011). Compact passively self-tuning energy harvesting for rotating applications. Smart Mater. Struct..

[72] Tang Q.C., Xia X.Y., Li X.X. Non-contact frequency-up-conversion energy harvester for durable & broad-band automotive TPMS application.

[73] Wang Y.J., Chen C.D., Sung C.K. (2012). System Design of a Weighted-Pendulum-Type Electromagnetic Generator for Harvesting Energy From a Rotating Wheel. IEEE/ASME Trans. Mechat..

[74] Keck M. A new approach of a piezoelectric vibration-based power generator to supply next generation tire sensor systems.

[75] Hatipoglu G., Urey H. (2009). FR4-based electromagnetic energy harvester for wireless tyre sensor nodes. Procedia Chem..

[76] Suzuki Y. (2011). Recent progress in MEMS electret generator for energy harvesting. IEEE Trans. Electr. Electr. Eng..

[77] Chen Y.-Y., Pan H.-W. A Piezoelectric Vibration Energy Harvester for Tire Pressure Monitoring Systems.

[78] Luigi P., Maurizio V., Bo G.M. Experimental results of piezoelectric bender generators for the energy supply of smart wireless sensors.

[79] Tornincasa S., Repetto M., Bonisoli E., Monaco F.D. (2012). Energy harvester for vehicle tires: Nonlinear dynamics and experimental outcomes. J. Intell. Mater. Syst. Struct..

[80] Farbod K., Siamak A. (2008). Energy Harvesting from Pneumatic Tires Using Piezoelectric Transducers. ASME Conf. Proc..

[81] Tsujiuchi N., Koizumi T., Oshibuchi A., Shima I. Rolling Tire Vibration Caused by Road Roughness.

[82] Clark S.K., Dodge R.N. (1979). A handbook for the rolling resistance of pneumatic tires.

[83] Gusakov I. (1977). Laboratory Measurements of Tire Rolling Resistance under Simulated Driving Cycles.

[84] Wilburn D.K. (1972). A temperature study of pneumatic tires during highway operation.

[85] Narasimha K.V., Rao R.K., Kumar P.C., Mukhopadhyay R. (2006). A Finite Element Algorithm for the Prediction of Steady-State Temperatures of Rolling Tires. Tire Sci. Technol..

[86] Yeong-Jyh L., Sheng-Jye H. (2004). Temperature prediction of rolling tires by computer simulation. Math. Comput. Simul..

[87] Zepeng W. Finite Element Analysis of Mechanical and Temperature Field for a Rolling Tire.

[88] Ebbott T.G., Hohman R.L., Jeusette J.â.P., Kerchman V. (1999). Tire Temperature and Rolling Resistance Prediction with Finite Element Analysis. Tire Sci. Technol..

[89] Bouendeu E., Greiner A., Smith P.J., Korvink J.G. (2011). A Low-Cost Electromagnetic Generator for Vibration Energy Harvesting. IEEE Sens. J..

[90] Saha C.R., O'Donnell T., Loder H., Beeby S., Tudor J. (2006). Optimization of an Electromagnetic Energy Harvesting Device. IEEE Trans. Magnet..

[91] Beeby S.P., O'Donnell T., Priya S., Inman D.J. (2009). Electromagnetic Energy Harvesting. Energy Harvesting Technologies.

[92] Amirtharajah R., Chandrakasan A.P. (1998). Self-powered signal processing using vibration-based power generation. IEEE J. Solid-State Circuit..

[93] Stephen B., White N. (2010). Chapter 4 Kinetic Energy Harvesting. Energy Harvesting for Autonomous Systems.

[94] Manoli Y. Energy harvesting–from devices to systems.

[95] Khan S.F.U. (2011). Vibration-based Electromagnetic Energy Harvesters for MEMS Applications. Ph.D. Thesis.

[96] Beeby S., Tudor M., White N. (2006). Energy harvesting vibration sources for microsystems applications. Measurem. Sci. Technol..

[97] Trainer M. (2003). Kelvin and piezoelectricity. European J. Phys..

[98] Yang J. (2005). Chapter one, Sec. 2.2 Piezoelectric effect. An introduction to the theory of piezoelectricity.

[99] Woias P. Micro energy harvesting as a core technology for energy-autonomous embedded systems.

[100] Chew Z., Li L. (2010). Design and characterisation of a piezoelectric scavenging device with multiple resonant frequencies. Sens. Actuators A Phys..

[101] Kok S.L. (2010). Design, Fabrication and Characterisation of Freestanding Thick-Film Piezoelectric Cantilevers for Energy Harvesting. Ph.D. Thesis.

[102] Advanced Cerametrics Piezo Fibre Composite transducer. http://www.advancedcerametrics.com/.

[103] Bogue R. (2009). Energy harvesting and wireless sensors: A review of recent developments. Sens. Rev..

[104] Churchill D.L.H., Michael J.T., Christopher P., Arms S.W. Strain energy harvesting for wireless sensor networks.

[105] Steven C.P.T., Arms W., David L.C. Energy Harvesting Wireless Sensors for Helicopter Damage Tracking.

[106] Yang B., Lee C., Kee W.L., Lim S.P. (2010). Hybrid energy harvester based on piezoelectric and electromagnetic mechanisms. J. Micro/Nanolithogr. MEMS MOEMS.

[107] Qin Y., Wang X., Wang Z.L. (2008). Microfibre-nanowire hybrid structure for energy scavenging. Nature.

[108] Vinod R.C., Prasad M.G., Fisher F.T. (2009). A coupled piezoelectric–electromagnetic energy harvesting technique for achieving increased power output through damping matching. Smart Mater. Struct..

[109] Kim H., Tadesse Y., Priya S., Priya S., Inman D.J. (2009). Piezoelectric Energy Harvesting Energy Harvesting Technologies.

[110] Qi Y., Jafferis N.T., Lyons K., Lee C.M., Ahmad H., McAlpine M.C. (2010). Piezoelectric Ribbons Printed onto Rubber for Flexible Energy Conversion. Nano Lett..

[111] Min G. (2010). Chapter 5: Thermoelectric Energy Harvesting. Energy Harvesting for Autonomous Systems.

[112] Shu Y.-C. (2009). Chapter 3: Performance Evaluation of Vibration-Based Piezoelectric Energy Scavengers. Energy Harvesting Technologies.

[113] Pereyma M. Overview of the Modern State of the Vibration Energy Harvesting Devices.

[114] Mitcheson P.D., Reilly E.K., Toh T., Wright P.K., Yeatman E.M. (2007). Performance limits of the three MEMS inertial energy generator transduction types. J. Micromech. Microeng..

[115] (1988). IEEE Standard on Piezoelectricity.

[116] Steve C.L.Y. (2004). Low Power Wireless Sensor Applications, in Department of Computer Science & Engineering. Master Thesis.

[117] Hu J., Yuan F.-G., Xu F., Huang A.Q. An optimal design of magnetostrictive material (MsM) based energy harvester.

[118] Cammarano A., Burrow S.G., Barton D.A.W., Carrella A., Clare L.R. (2010). Tuning a resonant energy harvester using a generalized electrical load. Smart Mater. Struct..

[119] Clare L.R., Burrow S.G. Power conditioning for energy harvesting.

[120] Ramond A., Sanchez M., Li K., Durou H., Jammes B., Rossi C. A single inductor dido converter with ultra low power mppt and thin-film lipon battery for piezoelectric energy harvesting and management.

[121] Guan M.J., Liao W.H. (2008). Characteristics of Energy Storage Devices in Piezoelectric Energy Harvesting Systems. J. Intell. Mater. Syst. Struct..

[122] Sodano H.A., Inman D.J., Park G. (2005). Comparison of Piezoelectric Energy Harvesting Devices for Recharging Batteries. J. Intell. Mater. Syst. Struct..

[123] Squires D. Overview of Storage Technologies for Energy Harvesting Applications.

[124] Ludvigsen K. (2003). Porsche: Excellence Was Expected.

[125] Herndl T. (2010). Remote Sensing of Car Tire Pressure. Energy Harvesting Systems: Principles, Modeling and Applications.

[126] Matsuzaki R., Todoroki A. (2008). Wireless Monitoring of Automobile Tires for Intelligent Tires. Sensors.

[127] Lemon Law Center TPMS Legislation. http://lemon.onecle.com/fmvss-standard-no-138/.

[128] Blaney J.R., Benoit W.L., Brazeal L.M. (2002). Blowout! Firestone's image restoration campaign. Public Relat. Rev..

[129] Uemura Y., Oshita S., Konno T., Kobuna T., Yoshida Y. (1985). Tire Pressure Warning System. SAE Techn. Papers.

[130] Qi Z., Bo L., Li G.F. Design of tire pressure monitoring system based on resonance frequency method.

[131] Thiriez K.K. (2006). Evaluation of indirect tire pressure monitoring systems using data from NCSA's tire pressure special study. US National Highway Traffic Safety Administration.

[132] APOLLO, Intelligent Tyre Systems–State of the Art and Potential Technologies; IST-2001–34372.

[133] Flatscher M., Dielacher M., Herndl T., Lentsch T., Matischek R., Prainsack J., Pribyl W., Theuss H., Weber W. (2010). A Bulk Acoustic Wave (BAW) Based Transceiver for an In-Tire-Pressure Monitoring Sensor Node. IEEE J. Solid-State Circui..

[134] Marsh D. Safety check, wireless sensors eye tyre pressure. http://www.edn.com/file/15794–421537.pdf.

[135] Lange T., Löhndorf M., Kvisterøy T. (2007). Intelligent Low-Power Management and Concepts for Battery-less Direct Tire Pressure Monitoring Systems (TPMS). Advanced Microsystems for Automotive Applications 2007.

[136] Becker J., Krueger S., Gessner W. (2002). Tire Pressure Monitoring Systems—the New MEMS Based Safety Issue Advanced Microsystems for Automotive Applications Yearbook 2002.

[137] Cohen P., Valldorf J., Gessner W. (2003). VisiTyre: A TPMS Solution Employing Directly Connected, 2-Wire Communication Channel to Achieve Highly Predictable and Reliable Performance. Advanced Microsystems for Automotive Applications 2003.

[138] Kurashige T., Kobane S., Takahashi H., Komatsu S. (1982). Low Tire Pressure Warning Device (LTPWD). SAE Techn. Papers.

[139] Dixon B., Kalinin V., Beckley J., Lohr R. A Second Generation In-Car Tire Pressure Monitoring System Based on Wireless Passive SAW Sensors.

[140] Schimetta G., Dollinger F., Weigel R. (2000). A wireless pressure-measurement system using a SAW hybrid sensor. IEEE Trans. Microw. Theory Techn..

[141] Deicke F., Graetz H., Fischer W.-J. Analysis of Antennas for Sensor Tags Embedded in Tyres.

[142] Nabipoor M., Majlis B.Y. (2006). A new passive telemetry LC pressure and temperature sensor optimized for TPMS. J. Phys. Confer. Series.

[143] Ho I.H., Jia-Min C., Hsiao-Chin C., Hung-Wei C. A Battery-Less Tire Pressure Monitoring System.

[144] Gruber S., Reinisch H., Unterassinger H., Wiessflecker M., Hofer G., Klamminger M., Pribyl W., Holweg G. A passively powered BAW-based multi-standard in-tire identification and monitoring system.

[145] Grossmann R. Wireless measurement of tire pressure with passive quartz sensors.

[146] Flatscher M., Dielacher M., Prainsack J., Matischek R., Herndl T., Lentsch T., Pribyl W. (2008). A bulk acoustic wave(BAW)-based sensor node for automotive wireless sensor networks. e & i Elektrotechnik und Informationstechnik.

[147] Vijay K., Varadan K., Jose A., Varadan V.V. (2001). Design and development of passive MEMS-IDT sensors for continuous monitoring of tire pressure.

[148] Makki N., Pop-Iliev R. (2012). Battery-and wire-less tire pressure measurement systems (TPMS) sensor. Microsyst. Technol..

[149] Makki N., Pop-Iliev R. Piezoelectric power generation for sensor applications: design of a battery-less wireless tire pressure sensor.

[150] Makki N., Pop-Iliev R. Pneumatic tire-based piezoelectric power generation.

[151] Roundy S. Energy Harvesting for Tire Pressure Monitoring Systems.

[152] Pinna L. (2010). Vibration-Based Energy Scavenging for Power Autonomous Wireless Sensor Systems, in School of Science and Technology for Information and Knowledge. Ph.D. Thesis.

[153] Frey A., Seidel J., Schreiter M., Kuehne I. Piezoelectric MEMS energy harvesting module based on non-resonant excitation.

[154] Hu Y., Xu C., Zhang Y., Lin L., Snyder R.L., Wang Z.L. (2011). A Nanogenerator for Energy Harvesting from a Rotating Tire and its Application as a Self-Powered Pressure/Speed Sensor. Adv. Mater..

[155] Gu L., Livermore C. Pendulum-driven passive self-tuning energy harvester for rotating applications.

[156] Vickery P.E., Nowell A. (2010). Post assembly automatic adjustment of TPMS sensor preload.

[157] Franks N.A., Franks M. (2011). System for accurately measuring and regulating air pressure in tires.

[158] Gao Z., Sham M.-L., Chung C.H. (2009). Piezoelectric Module for Energy Harvesting, Such as in a Tire Pressure Monitoring System.

[159] Blanchard R.A. (2010). Self-Powered Sensor System for Monitoring Tire Pressure.

[160] Bouchaud J., Dixon R. Market Prospects for Micro Power Technologies, iSuppli.

[161] Haswell S.W.F.G., Holdsworth P.R., Bowles S.J., Smart D.M., Garcia-Hernandez M.J., Chavez-Dominguez J.A., Turo-Peroy A., Salazar-Soler J. (2009). Power generator.

[162] (2010). Acts adopted by bodies created by international agreements. Official Journal of the European Union.

[163] Tuononen A.J. (2008). Optical position detection to measure tyre carcass deflections. Veh. Syst. Dyn..

[164] Anghelache G., Moisescu R., Sorohan S., Buretea D. (2011). Measuring system for investigation of tri-axial stress distribution across the tyre-road contact patch. Measurement.

